# The α-tubulin of *Laodelphax striatellus* mediates the passage of rice stripe virus (RSV) and enhances horizontal transmission

**DOI:** 10.1371/journal.ppat.1008710

**Published:** 2020-08-20

**Authors:** Yao Li, Danyu Chen, Jia Hu, Kun Zhang, Lin Kang, Yan Chen, Lijun Huang, Lu Zhang, Yin Xiang, Qisheng Song, Fang Liu

**Affiliations:** 1 College of Horticulture and Plant Protection, Yangzhou University, Yangzhou, China; 2 Jiangsu Co-Innovation Center for Modern Production Technology of Grain Crops, Yangzhou University, Yangzhou, China; 3 State Key Laboratory for Biology of Plant Diseases and Insect Pests, Institute of Plant Protection, Chinese Academy of Agricultural Sciences, Beijing, China; 4 Division of Plant Sciences, University of Missouri, Columbia, Missouri, United States of America; University of California, UNITED STATES

## Abstract

*Rice stripe virus* (RSV, genus *Tenuivirus*, family *Phenuiviridae*) is the causal agent of rice stripe disease transmitted by the small brown planthopper (SBPH, *Laodelphax striatellus*) in a persistent propagative manner. The midgut and salivary glands of SBPH are the first and last barriers to the viral circulation and transmission processes, respectively; however, the precise mechanisms used by RSV to cross these organs and transmit to rice plants have not been fully elucidated. We obtained the full-length cDNA sequence of *L*. *striatellus α-tubulin 2* (*LsTUB*) and found that RSV infection increased the level of LsTUB *in vivo*. Furthermore, LsTUB was shown to co-localize with RSV nonstructural protein 3 (NS3) *in vivo* and bound NS3 at positions 74–76 and 80–82 *in vitro*. Transient gene silencing of *LsTUB* expression caused a significant reduction in detectable RSV loads and viral *NS3* expression levels, but had no effect on NS3 silencing suppressor activity and viral replication in insect cells. However, suppression of LsTUB attenuated viral spread in the bodies of SBPHs and decreased RSV transmission rates to rice plants. Electrical penetration graphs (EPG) showed that LsTUB knockdown by RNAi did not impact SBPH feeding; therefore, the reduction in RSV transmission rates was likely caused by a decrease in viral loads inside the planthopper. These findings suggest that LsTUB mediates the passage of RSV through midgut and salivary glands and leads to successful horizontal transmission.

## Author summary

Over 70% of all known plant viruses are transmitted by specific arthropods, mainly including planthoppers, leafhoppers, aphids and whiteflies. Plant viruses with persistent relationships must overcome multiple barriers. Among these barriers, midgut and salivary glands are first and last barriers to the viral transmission. The interactions of host factors and virus in the insect midguts and salivary glands provide critical information regarding viral spread in the insect vector and subsequent infection of host plants. However, the precise mechanisms is still lacking. Here, we found that the specific interaction between a α-tubulin 2 of *Laodelphax striatellus* (SBPH) (LsTUB) and a nonstructural protein 3 (NS3) *Rice stripe virus* (RSV) mediates the passage of RSV through midgut and salivary glands, thereby leading to successful horizontal transmission. Our results confirm novel functions of LsTUB and NS3 in RSV transmission in insect vector. LsTUB may be a promising target for blocking horizontal transmission of RSV. These insights provide a better understanding of the interaction between plant viruses and vectors and may develop novel methods to control the systemic spread of plant viruses.

## Introduction

The survival of plant viruses is largely dependent on the efficient transmission to plant hosts by virus-specific vectors [1, 2]. Over 70% of all known plant viruses are transmitted by insects, and approximately 55% are vectored by Hemipteran insects (e.g. leafhoppers, planthoppers, aphids and whiteflies) [3]. These insects have distinctive piercing-sucking mouthparts with needle-like stylet bundles that are comprised of two maxillary and two mandibular stylets, making insect more suitable for virus transmission [3–5]. Four categories of insect vector—plant virus transmission relationships have been described as follows: nonpersistent; semipersistent; persistent circulative and persistent propagative [2, 6]. Plant viruses with persistent relationships enter vectors via the alimentary canal during feeding. After traversing several key tissues, these viruses eventually enter the salivary glands, which consist of principal and accessory glands. Furthermore, some even enter ovaries, which is another type of terminal insect tissue. In the principal salivary glands, viral particles are released into insect saliva when the vector is probing the host plant. In the ovaries, viruses are vertically transmitted to offspring [7]. Barriers to the persistent transmission of plant viruses in insect vectors include the following: (i) midgut infection barriers; (ii) dissemination barriers, including midgut escape and salivary gland infection barriers; (iii) salivary gland escape barriers; and (iv) transovarial transmission barriers [8, 9]. A deeper understanding of the mechanistic basis of virus transmission through these four barriers will facilitate the development of novel methods to control the systemic spread of plant viruses.

Previous studies demonstrated that the persistent transmission of viruses in different insect tissues requires specialized interactions between components of the virus and vector [10]. For example, in the aphid *Myzus persicae*, the coat protein read-through domain (CP-RTD) of *Beet western yellows virus* bound Rack-1 and membrane-bound glyceraldehyde-3-phosphate dehydrogenase to facilitate transcytosis of luteoviruses in the aphid midgut and accessory salivary glands [11]. The coat proteins of *Tomato leaf curl New Delhi virus* and *Cotton leaf curl Rajasthan virus* were shown to interact with a *Bemisia tabaci* midgut protein to facilitate trafficking of viral particles from the midgut into the insect hemolymph [12]. Furthermore, the *Rice ragged stunt virus* nonstructural protein Pns10 interacted with the *Nilaparvata lugens* oligomycin-sensitivity conferring protein to enhance viral titer in salivary gland cells [13]. Such interactions in different insect vectors are highly complex and diverse, and their effects on the horizontal transmission of viruses remain unclear.

*Rice stripe virus* (RSV, genus *Tenuivirus* of the *Phenuiviridae* family) has inflicted severe yield losses in rice throughout East Asia [14, 15]. RSV is transmitted by the small brown planthopper (SBPH), *Laodelphax striatellus*, in a persistent, circulative-propagative manner. The genome of RSV consists of four single-stranded, negative sense (ambisense) RNA molecules encoding the following seven proteins: RNA-dependent RNA polymerase (RdRP), RNA silencing suppressor with an unknown function (NS2), putative membrane glycoprotein (NSvc2), RNA silencing suppressor (NS3), nucleocapsid protein (CP), nonstructural disease-specific protein (NS4) and movement protein (NSvc4) [16–22]. Among these proteins, nonstructural protein NS3 functions as a gene-silencing suppressor in plants and functions in the size-independent and noncooperative recognition of dsRNA in plants [22, 23]; however, the role of NS3 in the insect vector, SBPH, has not been elucidated.

RSV particles initially establish infection in the midgut epithelium, then disseminate to the midgut visceral muscles, and ultimately enter the SBPH salivary glands before the virus can be transmitted to rice plants along with saliva [24]. Recently, molecular interactions between RSV and various SBPH tissues have received increased attention [25]. In the midgut, a direct interaction between NS4 and RSV CP promoted viral spread in viruliferous SBPH [24]. Furthermore, the interaction between the SBPH sugar transporter 6 (LsST6) and RSV CP was shown to be essential for RSV transfer across the midgut infection barrier [26], whereas the interaction between RSV CP and SBPH vitellogenin (LsVg) facilitated vertical transmission of the virus [27]. Further work revealed that *LsVg* expression was tissue-specific, and that LsVg produced in hemocytes was responsible for vertical transmission of RSV [28]. We previously reported that RSV was horizontally transmitted to rice plants via salivation during the feeding of insect vectors [29]. A series of salivary gland-specific transcriptome and proteome analyses revealed numerous genes involved in RSV transmission [30, 31]; however, only cuticular protein (CPR1) and a G protein pathway suppressor 2 (GPS2) impacted RSV transmission and replication in salivary glands [31, 32]. Obviously, the mechanism that RSV uses to overcome the salivary gland barrier and then undergo horizontal transmission to the plant warrants further investigation.

In the present study, proteomic analysis revealed that α-tubulin (TUB) was highly expressed in viruliferous SBPH compared to non-infected SBPH, suggesting that *L*. *striatellus* TUB (LsTUB) may have a role in mediating RSV transmission. Our data show that LsTUB facilitated the passage of RSV through the midgut and salivary gland barriers and enhanced viral transmission from SBPH to rice plants. Yeast two-hybrid and pull-down assays provided evidence that the interaction of LsTUB and the RSV NS3 likely constitutes a critical step in RSV transmission.

## Results

### cDNA cloning and sequence analysis of *LsTUB*

**Fig 1:**
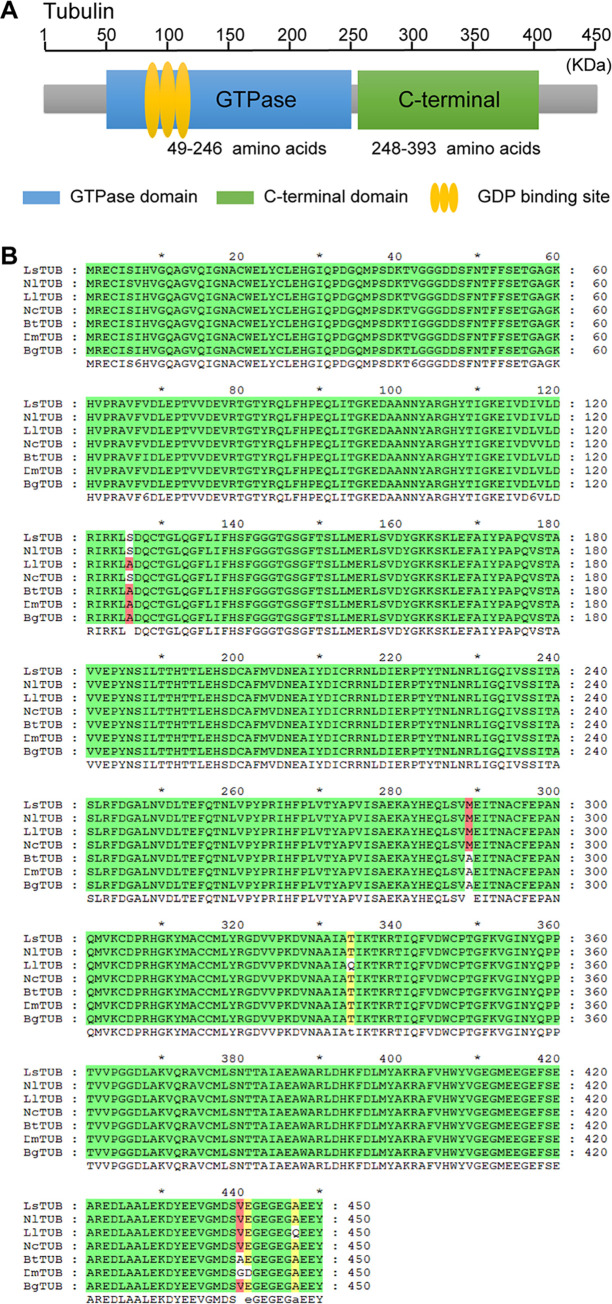
LsTUB protein structure and amino acid alignment. (A) Schematic representation of LsTUB. (B) Deduced amino acid sequence alignments of TUB in seven insect species; alignments were constructed using ClustalW software. Green shading indicates conserved tubulin residues in seven insect species; red or yellow shading indicates species-specific residues. Abbreviations indicate tubulin from the following insect species: LsTUB, *Laodelphax striatellus*; NlTUB, *Nilaparvata lugens*; LlTUB, *Lygus lineolaris*; NcTUB, *Nephotettix cincticeps*; BtTUB, *Bombus terrestris*; DmTUB, *Drosophila melanogaster*; and BgTUB, *Blattella germanica*.

Proteomic analysis of SBPH salivary glands identified 33 differentially expressed proteins in viruliferous and non-infected SBPH, including LsTUB (Table 1). Because tubulin heterodimers are known to function in viral assembly and transport [33–35], a full-length cDNA sequence of *LsTUB* (1,658 bp, GenBank accession no. KF934411) was cloned from female SBPH adults, using the conserved sequence of Tubulin α-2 from NCBI (GenBank accession no. AY550922.1) as an *in silico* probe. *LsTUB* contains a 1,353-bp open reading frame encoding a putative protein of 450 amino acids, a 93-bp 5’ untranslated region (UTR), and a 212-bp 3’ UTR. The translated cDNA of *LsTUB* yields a protein with a mass of approximately 50.0 kDA, and theoretical isoelectric point (pI) of 5.01. SMART analysis showed that LsTUB contains two conserved domains, including a GTPase domain (amino acids 49–246) with a GDP-binding site (amino acids 142–147) and a C-terminal domain (amino acids 248–393) (Fig 1A). Alignment of the LsTUB predicted protein sequence with other TUB proteins indicated a high level of identity with Hemipteran TUB proteins, including NlTUB in *N*. *lugens* (GenBank accession no. ANJ04673.1, 100% identity) and LlTUB in *Lygus lineolaris* (GenBank accession no. AHG54247.1, 99% identity) (Fig 1B).

**Table 1:** Differentially expressed proteins identified from proteomic analysis of salivary glands of viruliferous and non-infected SBPH.

Accession no.	Protein names	Gene names	Organism	Identity	score	Coverage (%)	Changes expression levels
P15577	NADH-ubiquinone oxidoreductase chain 2	*ND2*	*Paramecium tetraurelia*	26.0%	118	3.74	Up-regulated
P84050	Histone H4	*His4*	*Rhodnius prolixus*	100.0%	524	5.06	Up-regulated
Q9VTN3	Mitochondrial import inner membrane translocase subunit Tim13	*Tim13*	*Drosophila melanogaster*	76.0%	167	15.56	Up-regulated
P18101	Ubiquitin-60S ribosomal protein L40 precursor	*RpL40*	*Drosophila melanogaster*	100.0%	401	19.05	Up-regulated
Q9VTN3	Mitochondrial import inner membrane translocase subunit Tim13	*Tim13*	*Drosophila melanogaster*	76.0%	167	15.56	Up-regulated
Q9VTN3	Mitochondrial import inner membrane translocase subunit Tim13	*Tim13*	*Drosophila melanogaster*	76.0%	167	15.56	Up-regulated
Q9VTN3	Mitochondrial import inner membrane translocase subunit Tim13	*Tim13*	*Drosophila melanogaster*	76.0%	167	15.56	Up-regulated
Q4GXP3	Ribosomal protein S21		*Riptortus pedestris*	90.0%	267	19.77	Up-regulated
P06754	Tropomyosin 1		*Laodelphax striatella*	99.0%	316	28.95	Up-regulated
**P06605**	**Tubulin alpha-2 chain**	***Tubulin α-2***	***Drosophila melanogaster***	**100.0%**	**457**	**47.83**	**Up-regulated**
Q16P20	Probable citrate synthase 2, mitochondrial precursor		*Aedes aegypti*	87.0%	228	31.25	Up-regulated
G8CV15	Glyceraldehyde-3-phosphate dehydrogenase	*GAPDH3*	*Laodelphax striatella*	100.0%	168	17.72	Up-regulated
P27634	Elongation factor 1-alpha (EF-1-alpha) (Fragment)		*Rhynchosciara americana*	99.0%	445	14.63	Up-regulated
Q24251	ATP synthase subunit d, mitochondrial	*ATPsyn-d*	*Drosophila melanogaster*	46.0%	212	38.82	Up-regulated
Q1ZZQ2	ACYPI000034 protein	*ACYPI000034*	*Acyrthosiphon pisum*	71.0%	148	22.22	Up-regulated
Q9VTP4	60S ribosomal protein L10a-2	*RpL10Ab*	*Drosophila melanogaster*	85.0%	283	30.68	Down-regulated
P35381	ATP synthase subunit alpha, mitochondrial precursor	*blw*	*Drosophila melanogaster*	46.0%	320	13.92	Down-regulated
P06605	Tubulin alpha-3 chain	*Tubulin α-3*	*Drosophila melanogaster*	100.0%	457	60.87	Down-regulated
Q94514	Cytochrome c oxidase subunit 5A, mitochondrial	*CoVa*	*Drosophila melanogaster*	67.0%	269	48.24	Down-regulated
P62925	Eukaryotic translation initiation factor 5A	*eIF-5A*	*Spodoptera frugiperda*	78.0%	405	58.62	Down-regulated
Q9V3P0	Peroxiredoxin 1	*Jafrac1*	*Drosophila melanogaster*	79.0%	168	22.09	Down-regulated
P29310	14-3-3 protein zeta	*14-3-3zeta*	*Drosophila melanogaster*	98.0%	254	21.59	Down-regulated
Q94920	Voltage-dependent anion-selective channel	*porin*	*Drosophila melanogaster*	60.0%	252	39.29	Up-regulated
Q4UM09	NADH-quinone oxidoreductase subunit E	*nuoE*	*Rickettsia felis*	65.0%	280	16.05	Down-regulated
B0UE41	ATP synthase subunit alpha	*atpA*	*Methylobacterium sp*.	90.0%	315	13.92	Down-regulated
P06603	Tubulin alpha-1 chain	*Tubulin α-1*	*Drosophila melanogaster*	100.0%	457	47.83	Up-regulated
M9V250	Glutamine synthetase 2	*GS2*	*Nilaparvata lugens*	91.0%	300	39.29	Down-regulated
P35381	ATP synthase subunit alpha, mitochondrial precursor	*blw*	*Drosophila melanogaster*	90.0%	416	13.92	Down-regulated
P35381	ATP synthase subunit alpha, mitochondrial precursor	*blw*	*Drosophila melanogaster*	90.0%	416	13.92	Up-regulated
P35381	ATP synthase subunit alpha, mitochondrial precursor	*blw*	*Drosophila melanogaster*	90.0%	416	13.92	Up-regulated
Q6LHK5	NAD-dependent malic enzyme 2		*Acyrthosiphon pisum*	100.0%	815	2.84	Up-regulated
Q6LHK5	Malic enzyme		*Acyrthosiphon pisum*	100.0%	815	2.84	Up-regulated
Q7KN62	Transitional endoplasmic reticulum ATPase TER94	*TER94*	*Drosophila melanogaster*	80.0%	176	44.32	Down-regulated

### RSV infection in SBPH increases *LsTUB* expression

**Fig 2:**
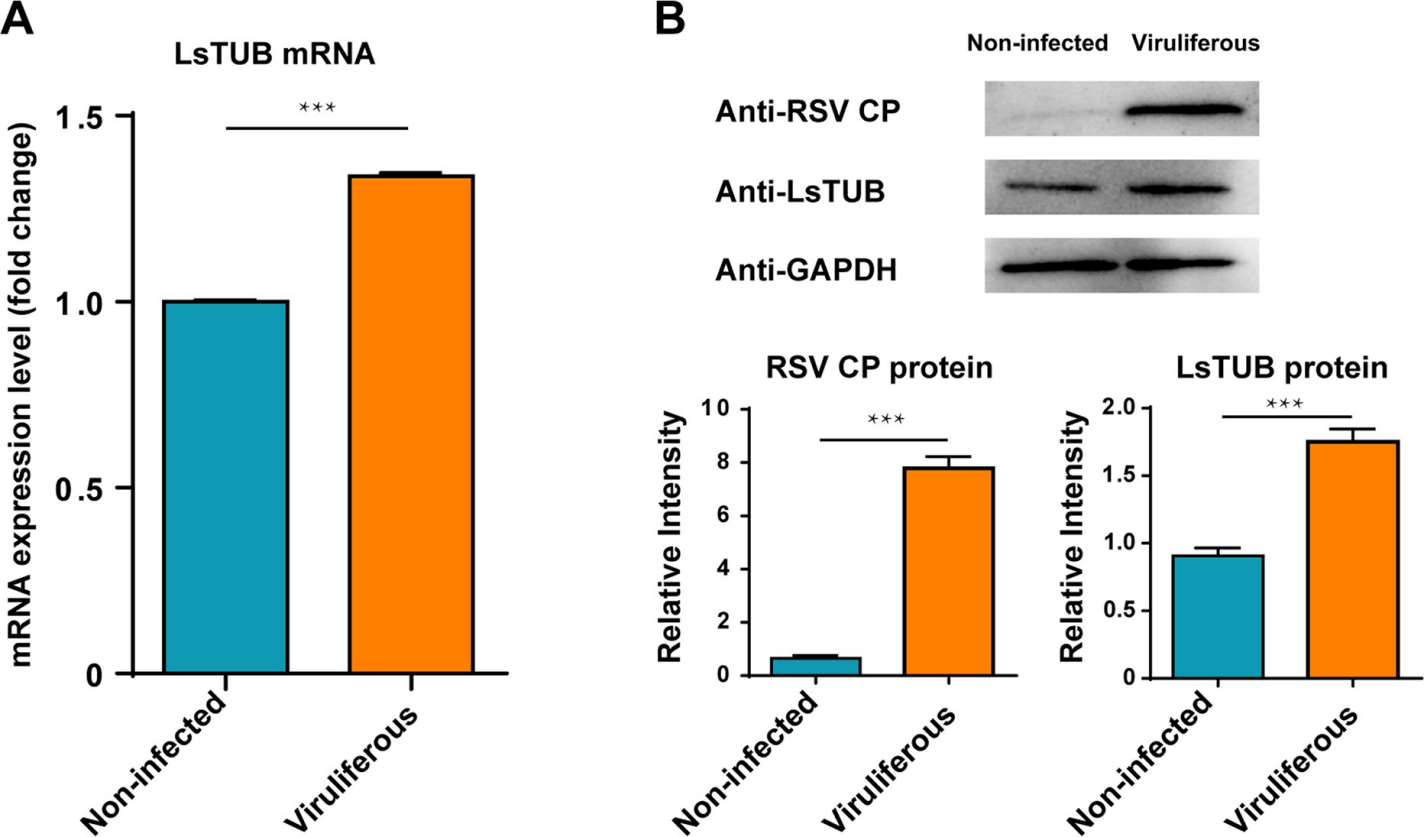
mRNA and protein expression levels of *LsTUB* in non-infected and viruliferous SBPH. (A) qRT-PCR analysis of *LsTUB* expression in 50 non-infected and RSV-infected SBPH adults. Treatments were replicated three times. (B) Western blot analysis of LsTUB production in 50 non-infected and viruliferous SBPH adults. GAPDH was used as control. Histogram in lower panel of (B) shows the relative intensity of bands in gels. Means ± SE, *t*-test analysis: ***P<0.001.

To further evaluate differential expression of *LsTUB* in viruliferous vs. non-infected SBPH, qRT-PCR and Western blot analyses were conducted to quantify its mRNA and protein expression levels, respectively. The mRNA expression levels of *LsTUB* were significantly up-regulated in viruliferous SBPH (Fig 2A). The trend in protein expression was consistent with changes in gene expression as determined by immunoblotting with anti-LsTUB antisera (Fig 2B, S1 Fig).

### LsTUB co-localizes with RSV in different SBPH tissues

**Fig 3:**
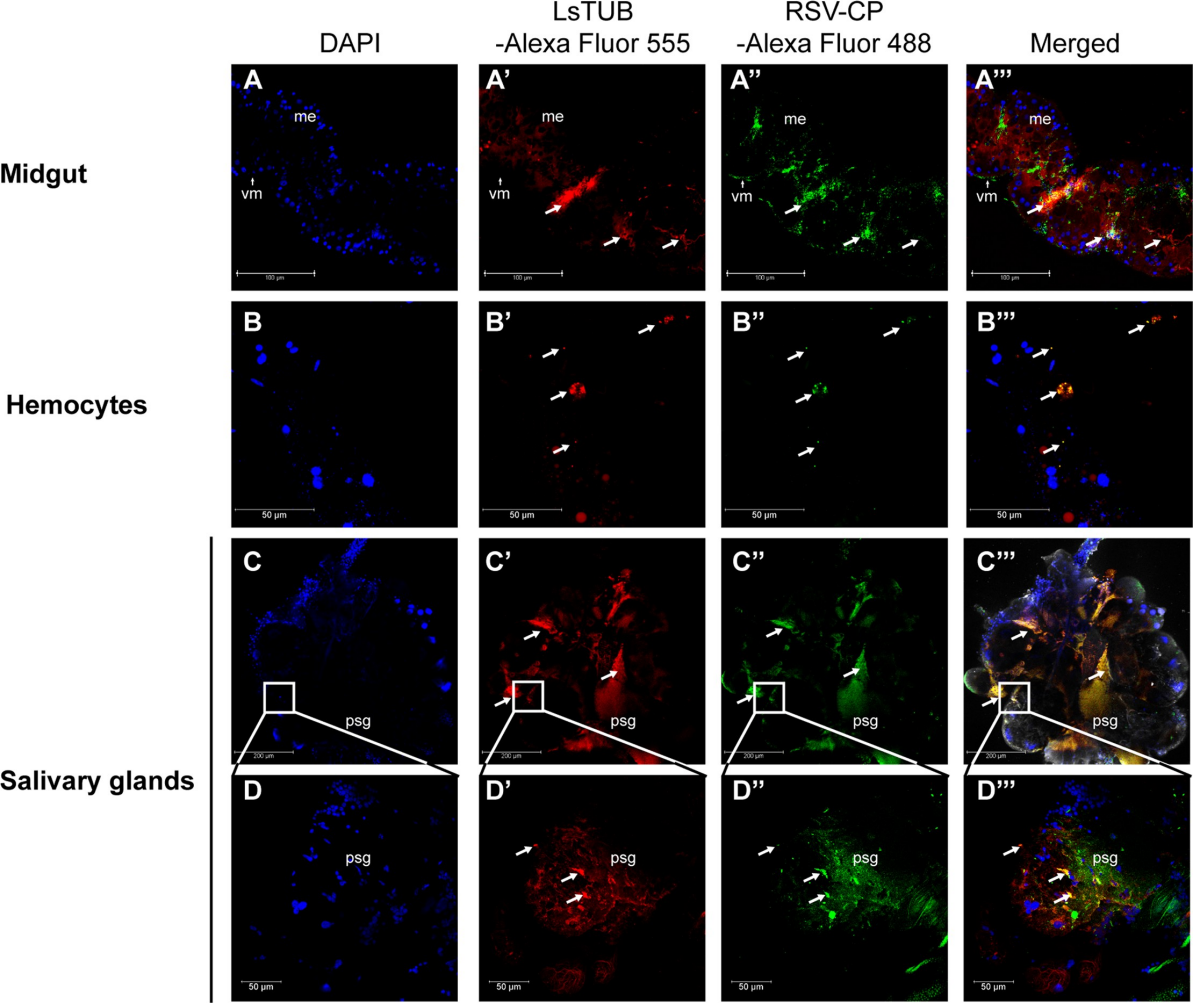
Localization of LsTUB and RSV in different tissues of SBPH. Detection of LsTUB antigen and RSV coat protein (CP) antigen in (A) midgut, (B) hemocytes, and (C) salivary glands of 30 viruliferous adults. Anti-LsTUB and anti-RSV CP monoclonal antibodies were detected using Alexa Fluor 555 (red) or 488 (green) labeled secondary antibodies, respectively. Panels A-A”’ and C-C”’, bar = 100 μm; panels B-B”’ and D-D”’, bar = 50 μm. Abbreviations: sg, salivary glands; psg, principal salivary glands; vm, visceral muscle; and me, midgut epithelium.

Since *LsTUB* was expressed at higher levels in response to RSV infection in viruliferous SBPH, we investigated whether they were co-localized in SBPH organs. Confocal imaging revealed that LsTUB and RSV CP were co-localized (see arrows, Fig 3A’–3D”’, S1 Table) in midgut (Fig 3A–3A”’), hemocytes (Fig 3B–3B”’) and principal salivary glands (Fig 3C–3C”’). The results indicate that LsTUB and RSV accumulate and co-localize throughout the SBPH body.

### LsTUB interacts with RSV NS3 *in vitro*

#### The interaction between LsTUB and RSV NS3

**Fig 4:**
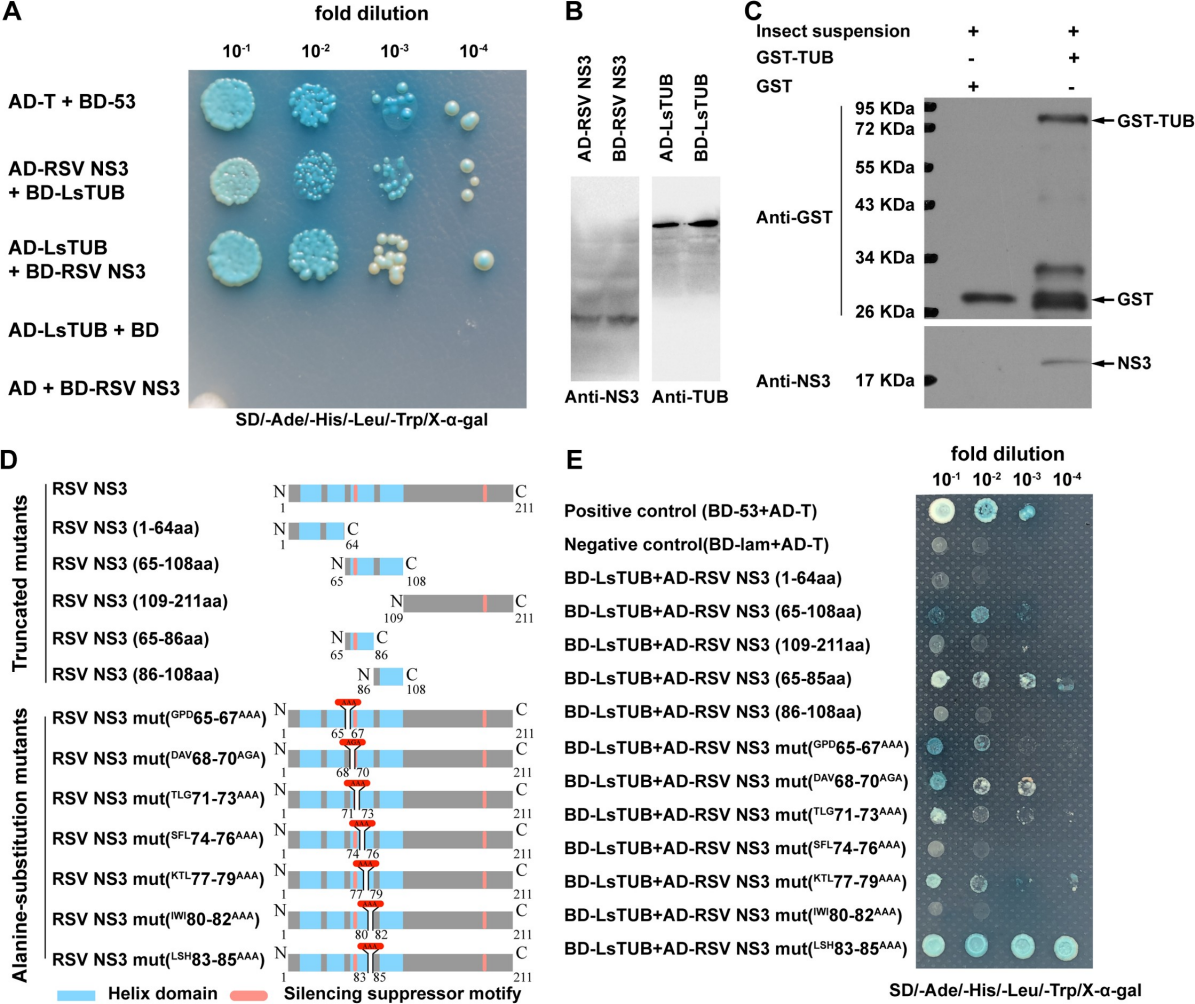
The interaction between LsTUB and RSV NS3 detected by yeast two-hybrid assay and GST pull-down assay. (A) Interactions between LsTUB and RSV NS3 proteins identified by yeast two-hybrid assays. Yeast cells were diluted 10^−1^ to 10^−4^ and plated onto QDO (SD-trp-leu-his-ade-20 mM3-AT) medium. Colonies growing on QDO were also assayed for β-galactosidase activity (blue color). Controls: AD-T + BD-53 (positive control); or AD-LsTUB + BD or AD + BD-RSV NS3 (negative control). Abbreviations: AD, activation domain, cloned in pGADT7; BD, bait domain, cloned in pGBKT7; LsTUB, *Laodelphax striatellus*, tubulin; NS3, nonstructural protein 3. (B) Western blot analysis of RSV NS3 and LsTUB in yeast strain Y2H Gold. (C) GST pull-down assays. LsTUB was fused to GST and incubated with viruliferous SBPH extracts or GST (control). Blots were probed with anti-NS3 or anti-GST antibodies. Insect suspension obtained from 50 viruliferous adults (femal/male ratio = 1:1), + means input,—means no input. (D) The schematic diagram of the wild-type RSV NS3 and 12 mutated RSV NS3 constructs. Five truncated mutants of NS3, including RSV NS3 1-64aa, RSV NS3 65-108aa, RSV NS3 109-211aa, RSV NS3 65-85aa and RSV NS3 86-108aa. Seven NS3 mutants with an alanine-substitution mutation, including RSV NS3 mut ^GPD^65-67^AAA^, RSV NS3 mut ^DAV^68-70^AGA^, NS3 mut ^TLG^71-73^AAA^, NS3 mut ^SFL^74-76^AAA^, NS3 mut ^KTL^77-79^AAA^, NS3 mut ^IWI^80-82^AAA^ and NS3 mut ^LSH^83-85^AAA^. (E) Interactions between LsTUB and mutated RSV NS3 proteins in yeast two-hybrid assays. Controls: AD-T + BD-53 (positive control) or AD-T + BD-Lam (negative control).

LsTUB was used as bait and a RSV cDNA library as prey in a yeast-two-hybrid (Y2H) assay designed to identify RSV proteins that potentially interact with LsTUB. After screening, RSV NS3 was found to interact with LsTUB in the Y2H assay (Fig 4A, S2 Fig). The expression of LsTUB and RSV NS3 in yeast was confirmed by Western blot analysis (Fig 4B). To further examine the interaction between LsTUB and RSV NS3, a pull-down assay was performed with glutathione S-transferase-tagged LsTUB (GST-TUB). When the extracts from viruliferous SBPH were incubated with GST-TUB, NS3 co-immunoprecipitated with GST-TUB as shown by Western blot analysis (Fig 4C).

#### The interaction between LsTUB and other viral proteins

Considering that other RSV proteins may also bind LsTUB, we evaluated whether LsTUB could interact with four RSV proteins (e.g. NSvc2, CP, SP and NSvc4) using Y2H analysis. Yeast strains containing full-length LsTUB as bait and each of the four proteins as prey failed to grow on synthetic dextrose dropout medium (S3 Fig). This result suggests that the interaction between LsTUB and NS3 is specific.

#### The binding site of LsTUB and RSV NS3

To determine the amino acid region of NS3 responsible for the interaction with LsTUB, we conducted Y2H analysis with three truncated mutants of NS3, including RSV NS3 1-64aa, RSV NS3 65-108aa, and RSV NS3 109-211aa (Fig 4D). Only one yeast strain harboring the truncated mutant RSV NS3 65-108aa and LsTUB grew, indicating a weak interaction (Fig 4E). The RSV NS3 65-108aa was then divided into the N-terminal fragment (RSV NS3 65-85aa) and C-terminal fragment (RSV NS3 86-108aa) (Fig 4D). Positive interactions existed between the RSV NS3 65-85aa and LsTUB (Fig 4E). Furthermore, seven NS3 mutants with an alanine-substitution mutation in the NS3 65-85aa region were generated and used to transform the yeast strain along with LsTUB (Fig 4D). When amino acid residues at positions 74–76 or positions 80–82 were substituted with alanine (RSV NS3 mut ^SFL^74-76^AAA^, RSV NS3 mut ^IWI^80-82^AAA^), the interaction between NS3 and LsTUB was abolished (Fig 4E). These results reveal that the amino acid residues at positions 74–76 and 80–82 of NS3 are required for the interaction between LsTUB and RSV NS3.

#### The interaction of LsTUB and RSV NS3 has no effect on silencing suppressor activity

**Fig 5:**
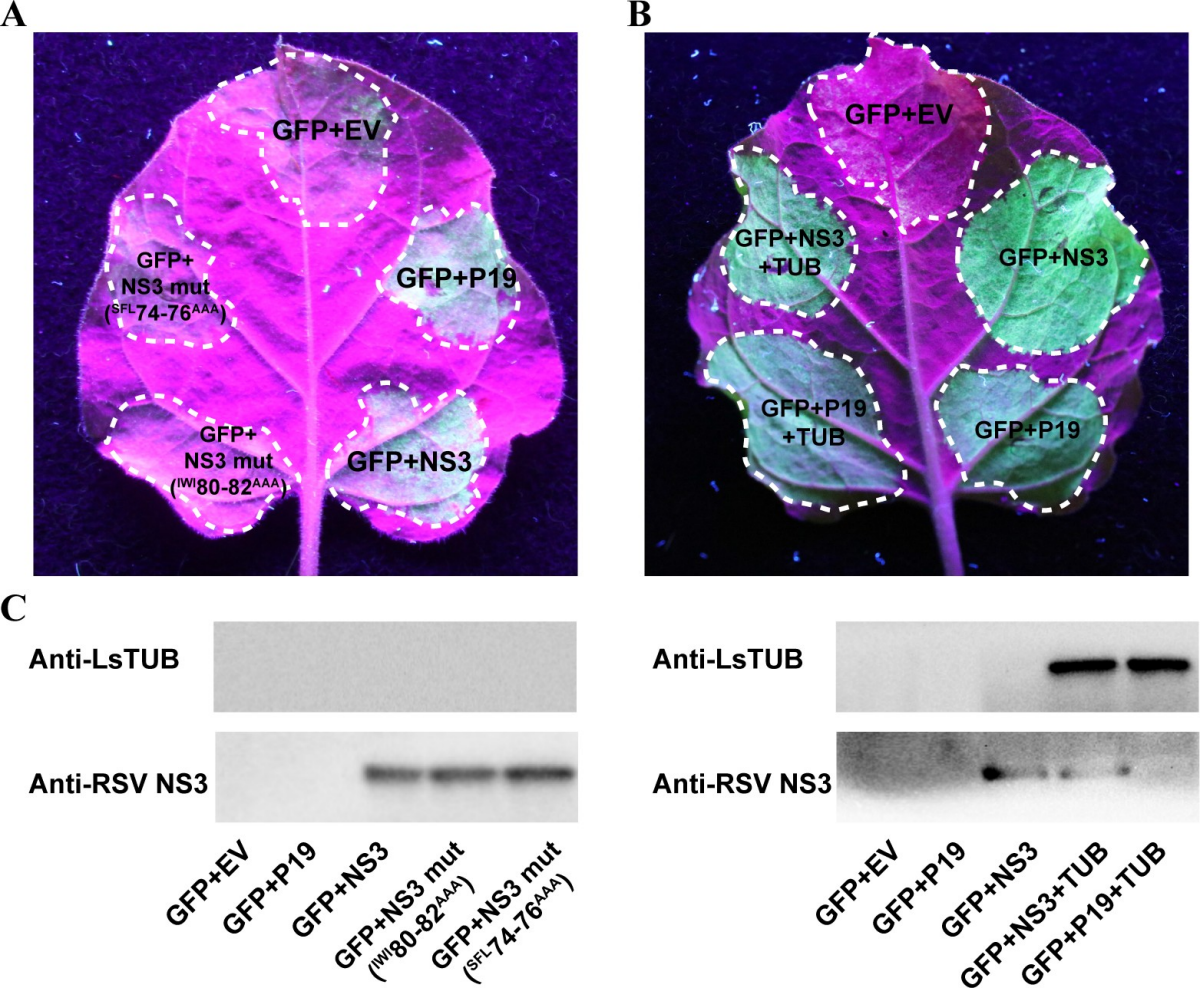
The effect of LsTUB on the RNA silencing suppressor activity of the RSV NS3 protein. (A) Leaves of wild-type *N*. *benthamiana* plants were co-agroinfiltrated with a GFP-expressing vector, a vector encoding dsGFP, an empty vector (EV), or a vector encoding P19 (positive control), or NS3, or NS3 mutants (NS3 mut ^SFL^74-76^AAA^ and NS3 mut ^IWI^80-82^AAA^). The leaves were photographed at three days post-infection under UV light. (B) Leaves of wild-type *N*. *benthamiana* plants were co-agroinfiltrated with a GFP-expressing vector, a vector encoding dsGFP, and an empty vector (EV), or a vector encoding P19 or NS3 (with or without a vector encoding LsTUB). (C) The expression of LsTUB and RSV NS3 in leaves from different treated spots was determined using anti-LsTUB and anti-RSV NS3 antibodies.

To determine whether the interaction of LsTUB and RSV NS3 would affect the latter’s silencing suppressor activity, we carried out a GFP (green fluoresce protein) silencing assay by transient ectopic expression of NS3 and its substitution mutants in *Nicotiana benthamiana* leaves. The results from the GFP silencing assay for the two mutants screened above showed that the substitutions of NS3 amino acids at positions 74–76 and 80–82 led to loss of their silencing suppressor ability as previously reported [36] (Fig 5A). However, leaves co-transfected with pBin438-NS3 and pBin438-LsTUB (see Methods) also exhibited high levels of GFP expression, and expression levels were similar to those in leaves transfected with pBin438-NS3 or the positive control (pBin438-P19) (Fig 5B). The expression of NS3 and LsTUB in *N*. *benthamiana* was verified by Western blot analysis (Fig 5C). These results clearly demonstrate that the presence of LsTUB has no effect on the silencing suppressor activity of RSV NS3 that would affect viral infection and accumulation.

### Silencing of *LsTUB* via RNAi reduces NS3 protein levels and RSV loads *in vivo*

To further explore the potential role of LsTUB in NS3-mediated transmission of RSV, 3^rd^ instar viruliferous SBPH nymphs were supplied with 0.5 mg/ml dsRNAs derived from *GFP* (dsGFP) or *LsTUB* (dsTUB) via membrane feeding. After seven days of feeding, qRT-PCR analysis showed that *LsTUB* mRNA in dsTUB-treated SBPHs was significantly reduced by more than 75% compared to the controls (untreated and dsGFP-treated SBPHs) (S4 Fig). These results indicate that RNAi-mediated knockdown of *LsTUB* is highly effective.

**Fig 6:**
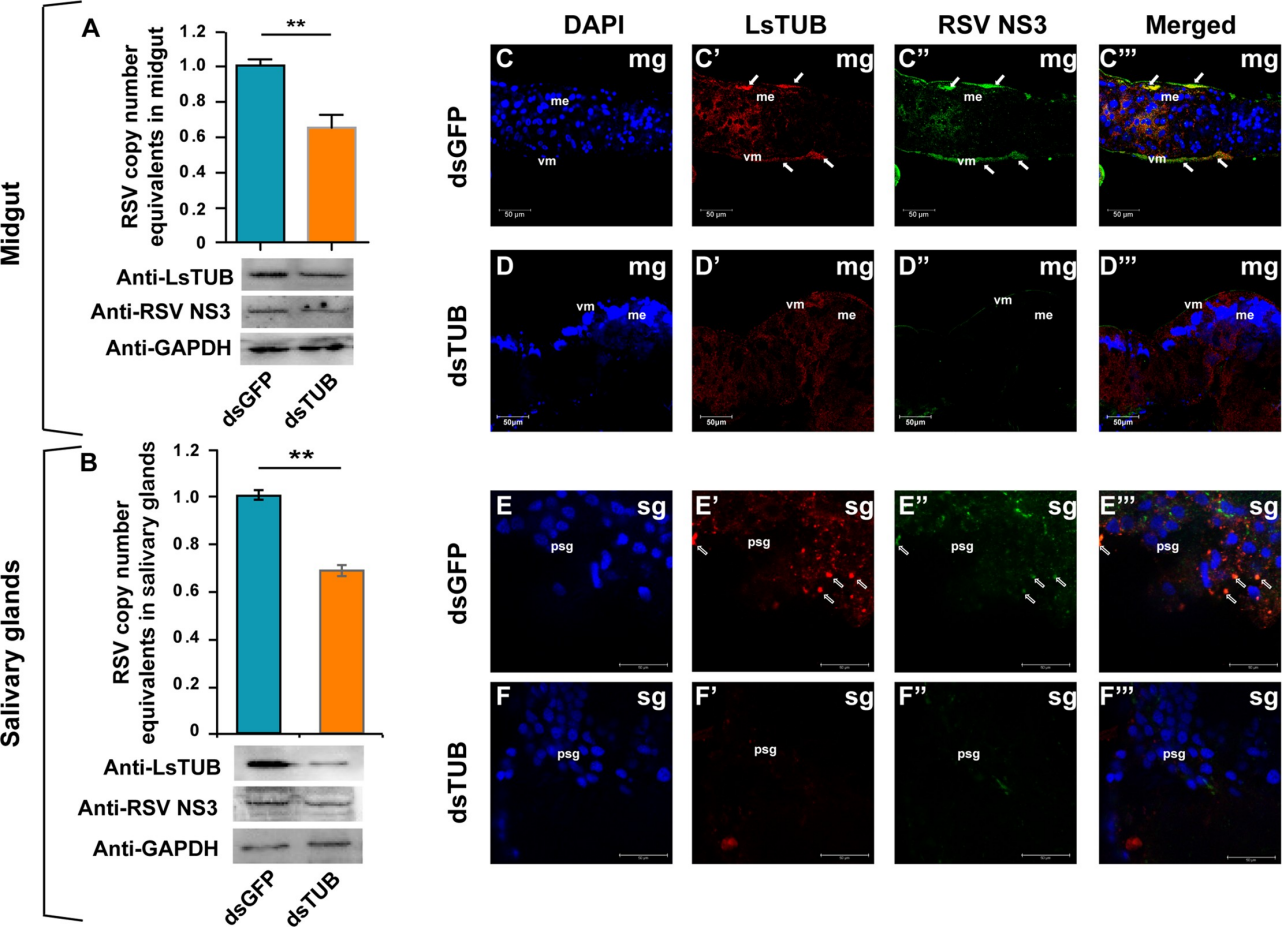
dsTUB-mediated RNAi inhibits RSV loads and reduces NS3 protein levels. Viruliferous SBPH were fed on artificial diets supplemented with dsGFP or dsTUB. SBPH midgut (A) and salivary glands (B) were analyzed for viral loads by qRT-PCR (red columns indicate dsTUB; blue columns indicate dsGFP). Levels of LsTUB, NS3 and GAPDH were analyzed by immunoblotting, and GAPDH was used as control. Each treatment was replicated three times for qRT-PCR, and values represent means ± SE, A student’s *t*-test was used to analyze significance; ** represents *P*<0.01. Midguts (Fig 5C–5D”’) and salivary glands (Fig 5E–5F”’) of viruliferous SBPH were immunolabeled with anti-RSV NS3 (Alexa Fluor 488, green) and anti-LsTUB (Alexa Fluor 555, red) and then examined by confocal microscopy. Abbreviations: mg, midgut; sg, salivary glands; and vm, visceral muscle. Bar = 50 μm.

The midgut and salivary glands of viruliferous SBPHs were also examined by immunoblotting and confocal microscopy. Immunoblotting showed that application of dsTUB led to a decrease in LsTUB in both midgut and salivary glands, accompanied by a decrease in RSV-NS3 (Fig 6A & 6B, S5 Fig). Immunofluorescence and qRT-PCR indicated that dsTUB treatment caused a substantial reduction in RSV loads in the midgut and salivary glands. Furthermore, LsTUB and NS3 were co-localized in tissues treated with dsGFP (see arrows), but not in tissues treated with dsTUB (Fig 6C–6F”’). Taken together, these results indicate that the interaction of LsTUB and NS3 is essential for RSV accumulation in the insect vector.

### Repression and overexpression of LsTUB do not affect RSV replication in SBPH cells

**Fig 7:**
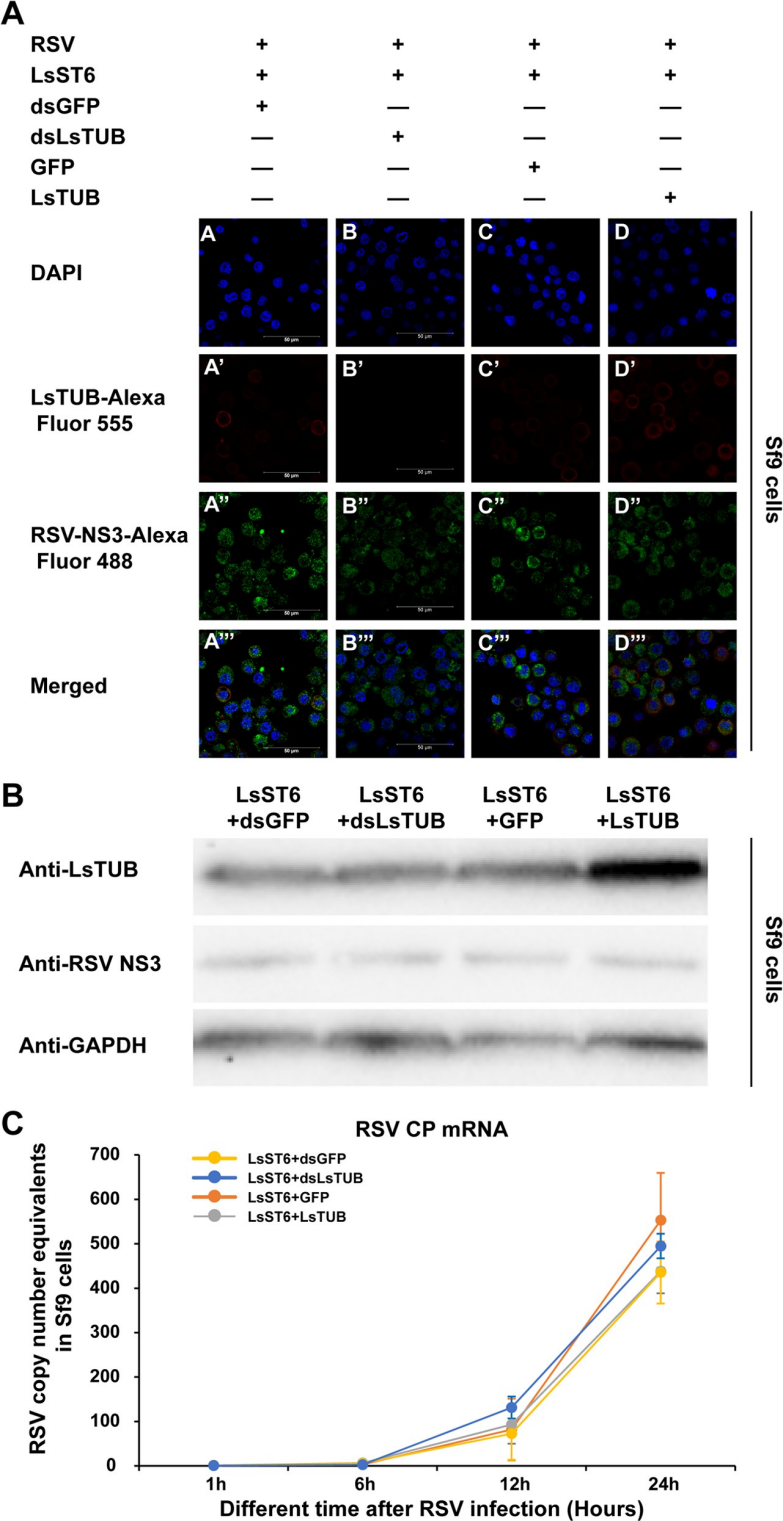
The effect of LsTUB overexpression and knockdown on RSV accumulation in LsST6 transfected Sf9 cells. (A) The effect of LsTUB overexpression and *LsTUB* knockdown on RSV NS3 in LsST6 transfected Sf9 cells at 24 h after RSV infection. The Sf9 cells were treated with anti-LsTUB antibody labeled with Alexa Fluor 555 (red) and anti-RSV NS3 antibody labeled with Alexa Fluor 488 (green) and then examined by confocal microscopy. Bars = 50 μm. (B) The protein expression level of LsTUB and RSV NS3 were analyzed by immunoblotting. GAPDH was used as control. (C) RSV copy number equivalents were tested by qRT-PCR in Sf9 cells. Total RNA was extracted from Sf9 cells at 1, 6, 12 and 24 h after adding virus particles to analyze the RSV loads. Values represent means ± SE; significance was evaluated by *t*-test analysis.

We examined whether the LsTUB-dependent decrease in RSV loads would affect viral replication in the insect. LsST6-expressing *Spodoptera frugiperda* 9 (Sf9) cells [26] were transfected with *LsTUB* and changes in RSV infection and replication were evaluated. At 1, 6, 12 and 24 h following RSV infection, RSV loads and NS3 protein levels were analyzed by immunofluorescence, Western blot and qRT-PCR in Sf9 cells in which LsTUB was silenced or overexpressed (Fig 7). Immunofluorescence analyses showed that NS3 co-localized with LsTUB in the Sf9 cell membrane, and stronger fluorescence signals of NS3 were detected in the cytoplasm of Sf9 cells at 24 h (Fig 7A). However, differential expression of *LsTUB* did not affect NS3 signal strength in Sf9 cells (Fig 7A). Western blot analysis showed that the transfection with LsTUB resulted in a significant increase of LsTUB mRNA abundance and protein levels, and treatment with dsLsTUB significantly reduced LsTUB expression compared to the controls (Fig 7B, S6 Fig). The RNA copy number equivalents of RSV were obviously 4 to 5-fold higher at 24 h than at 12 h post-RSV infection (Fig 7C). However, when the LsTUB was overexpressed or knocked down in Sf9 cells, the copy number equivalents of RSV RNA were not significantly reduced at 1, 6, 12 and 24 h post-infection compared to GFP RNA and dsGFP control treatments (Fig 7C). Taken together, these results suggest that overexpression or suppression of LsTUB does not significantly affect RSV replication in virus-infected insect cells.

### RNAi mediated silencing of LsTUB attenuates viral spread *in vivo*

**Fig 8:**
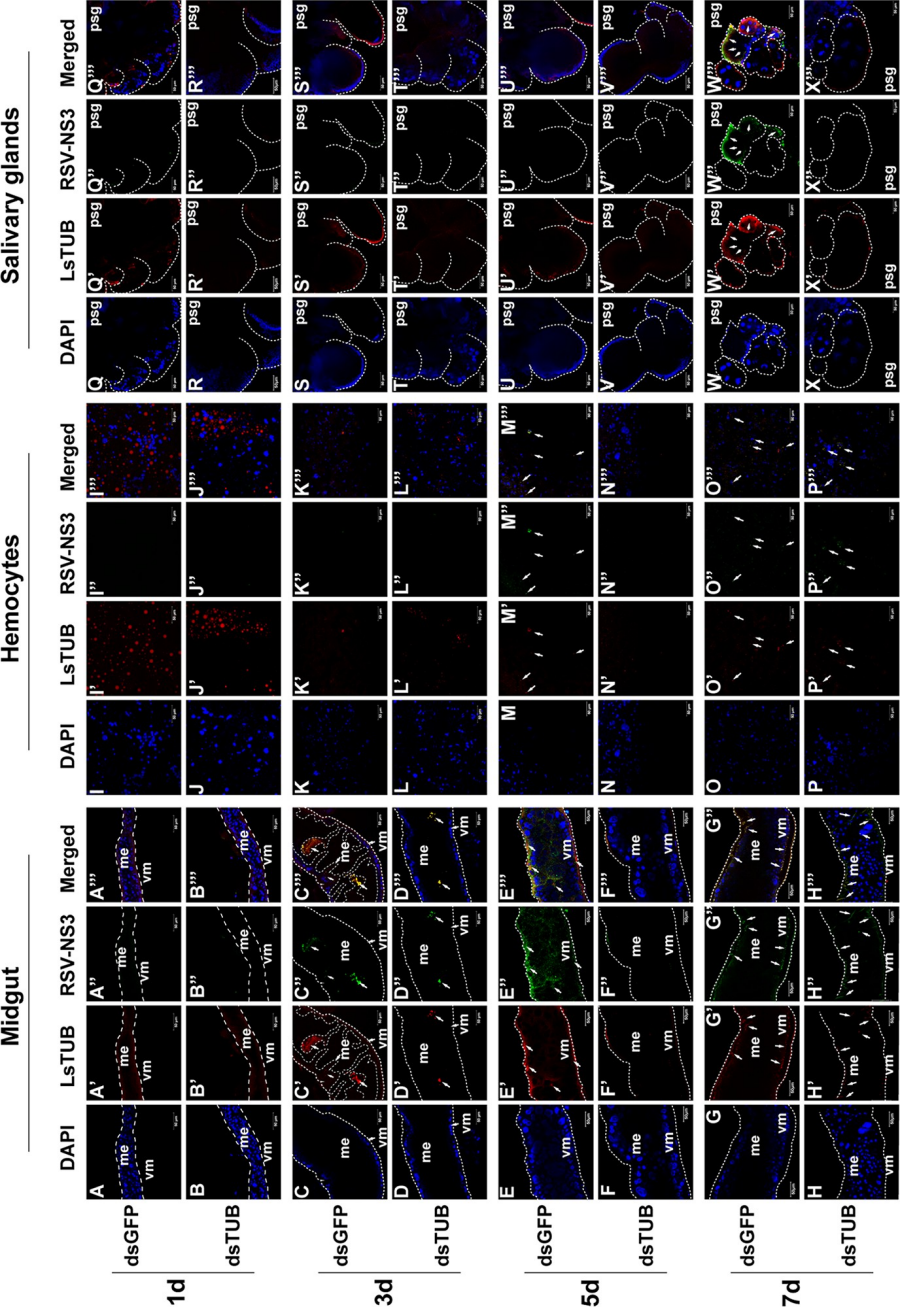
The effect of dsLsTUB on RSV spread in SBPH. Typical patterns of LsTUB and RSV NS3 distribution in different organs of dsGFP and dsLsTUB-treated SBPHs at 1 (A, B, I, J, Q, R), 3 (C, D, K, L, S, T), 5 (E, F, M, N, U, V), and 7 days padp (G, H, O, P, W, X). The midgut, hemocytes and salivary glands were labeled with anti-LsTUB antibody conjugated with Alexa Fluor 555 (red) and anti-RSV NS3 antibody conjugated with Alexa Fluor 488 (green) and then examined by confocal microscopy. Bars = 50 μm.

After eliminating the above possibilities, we proposed that LsTUB might play a critical role in the passage of RSV through midgut and salivary glands. We investigated the distribution of LsTUB and RSV NS3 in different organs from 400 dsGFP/dsLsTUB-treated non-infected SBPHs at 1, 3, 5, and 7 days after acquiring the virus from the infected plants. Once RSV was ingested by SBPH, the virus established its primary infection in the midgut epithelial cells and then spread to other insect tissues (Fig 8). At 3 days post-initial access to diseased plants (padp), viral infection was initially observed in a limited number of midgut epithelial cells in 14–15% of SBPHs treated with dsGFP or dsLsTUB (Table 2, Fig 8C–8C”’ & 8D–8D”’), suggesting that the treatment of dsLsTUB did not affect the early infection of RSV (S7 Fig). At 5 days padp, RSV spread to the visceral muscle cells surrounding the infected midgut epithelial cells in 22% of dsGFP-treated SBPHs (Table 2, Fig 8E–8E”’). Meanwhile, in 9% of the dsGFP-treated SBPHs, RSV NS3 antigen was also detected in the hemocyte cells. However, in 17% of dsLsTUB-treated insects, viral infection was essentially restricted in infection foci in the midgut with only very few hemocytes showing the presence of NS3 antigen (Table 2, Fig 8F–8F”’). At 7 days padp, RSV was found to accumulate in the entire alimentary canal in 31% of the dsGFP-treated SBPHs and spread to the salivary glands in 14% of the dsGFP-treated SBPHs (Table 2, Fig 8G–8G”’, 8O–8O”’ & 8W–8W”’). By contrast, only 3% of the dsLsTUB-treated SBPHs had infections in salivary glands (Table 2, Fig 8H–8H”’, 8P–8P”’ & 8X–8X”’). These results suggest that dsLsTUB treatment significantly attenuates RSV spread from the initial infection foci in the midgut to salivary glands.

**Table 2:** Infection of various SBPH tissues with plant-acquired RSV following silencing of LsTUB via dsRNA.

Treatment	Time (padp)	Number of positive SBPH with NS3 antigens detected by immunofluorescence microscopy at different days padp (*n* = 100)		
	Different tissues	Midgut	Hemocytes	Salivary glands
dsGFP	1 days	0	0	0
	3 days	15	0	0
	5 days	22	9	0
	7 days	31	29	14
dsLsTUB	1 days	0	0	0
	3 days	14	0	0
	5 days	17	4	0
	7 days	29	13	3

### Silencing of *LsTUB* via RNAi results in decreased RSV transmission efficiency

The ability of dsLsTUB-treated viruliferous SBPH to transmit RSV was evaluated. Following a two-day acquisition period on RSV-infected rice plants, each viruliferous SBPH treated with dsLsTUB or dsGFP was transferred to healthy rice seedlings, allowed to feed for two days, and then evaluated for virus infection rates by qRT-PCR (Table 3). At 15 days post-infection, 24.1% of rice plants fed on by dsLsTUB-treated SBPH contained RSV, compared to 61.3% of plants fed on by dsGFP-treated SBPH. The significance of transmission efficiency was evaluated by *χ^2^* analysis, and *P*-values indicated that the infection rate of dsLsTUB-treated SBPH was significantly lower than the dsGFP-treated viruliferous SBPH (Table 3).

**Table 3:** Transmission rates of viruliferous SBPH following LsTUB silencing via dsRNA.

Strains	Treatment	Virus transmission rates	*χ^2^*test		
			Treatments	*χ^2^*value	*P* value
Viruliferous SBPHs acquiring RSV from infected plant	dsGFP	61.3% (19/31)	dsTUB vs dsGFP	6.98	0.008
	dsTUB	24.1% (7/29)			
Viruliferous SBPHs acquiring RSV from parents	Control	40.0% (12/30)	Control vs dsTUB	4.07	0.044
	dsGFP	48.1% (13/27)	dsTUB vs dsGFP	6.36	0.012
	dsTUB	12.0% (3/25)	Control vs dsGFP	0.12	0.725

Furthermore, the effect of dsLsTUB on the transmission efficiency of SBPH acquiring RSV from parents was evaluated by qRT-PCR. At 15 days post-infection, about 12.0% of rice plants fed on by dsLsTUB-treated SBPH contained RSV, compared to over 40.0% of plants fed on by dsGFP-treated or untreated (control) SBPH (Table 3), indicating that RNAi-mediated knockdown of *LsTUB* inhibits horizontal transmission of the virus. These results indicate that LsTUB plays a function in RSV transmission from SBPH to rice plants.

### Silencing of *LsTUB* in viruliferous SBPH has no effect on its feeding behavior

**Fig 9:**
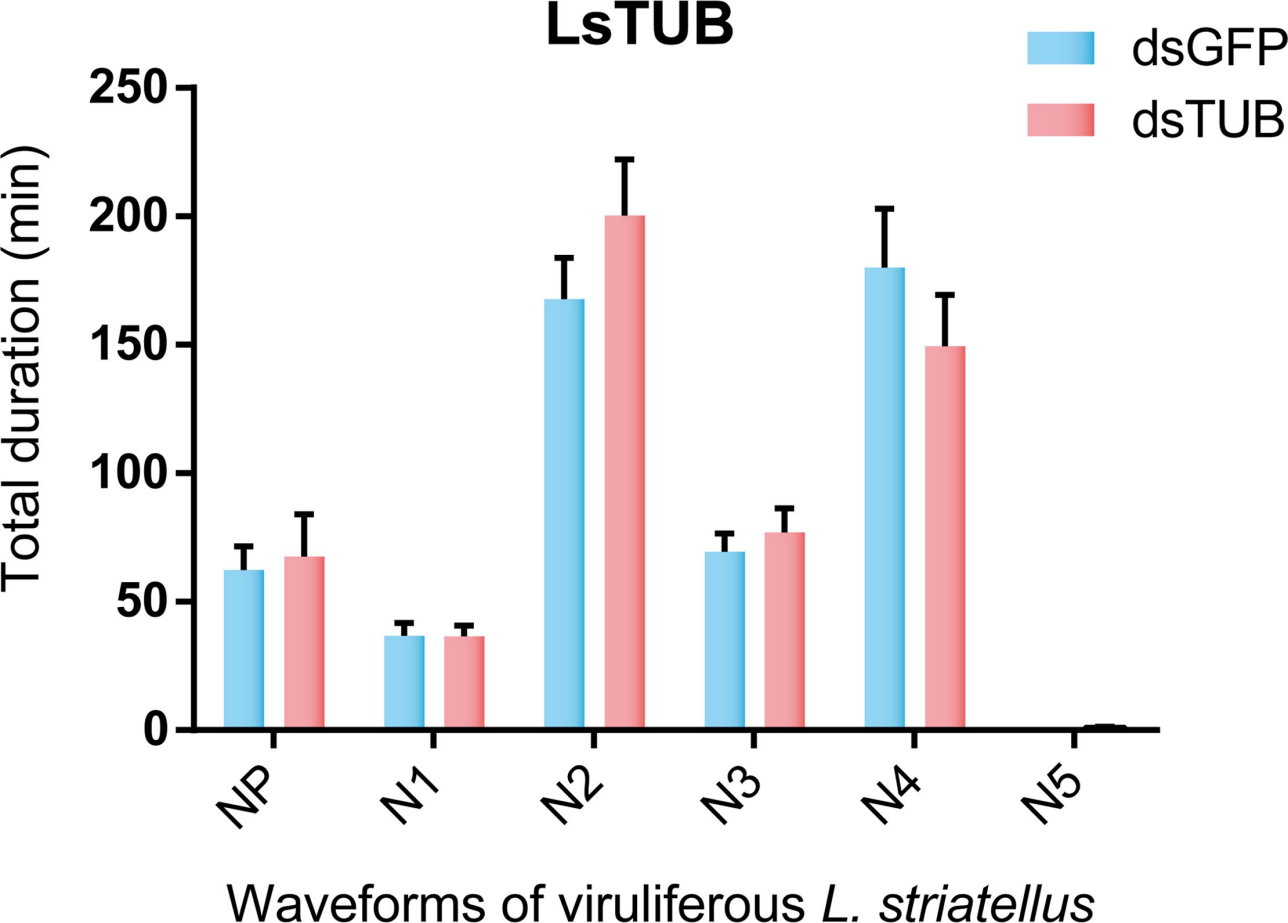
Electrical penetration graph (EPG) analysis of dsGFP or dsTUB-treated viruliferous *L*. *striatellus*. Waveform abbreviations: NP, non-penetration; N1, penetration; N2, salivation; N3, extracellular movement of stylet near the phloem; N4, sap ingestion in phloem; N5, water ingestion in xylem. Values represent means ± SE; significance was evaluated by *t*-test analysis.

To investigate whether the decrease in RSV transmission efficiency was caused by RNAi-mediated changes in SBPH feeding behavior, the electrical penetration graph (EPG) technique was used to monitor SBPH feeding [37]. EPG signals were classified into seven different waveforms including NP, N1, N2-a, N2-b, N3, N4, and N5, which represent the following phases: non-penetration, penetration, stylet movement with salivary secretion, sustained salivary secretion, extracellular movement of the stylet around the phloem, phloem feeding, and xylem feeding, respectively [29]. Representative EPG waveforms were not significantly different between dsGFP-treated and dsTUB-treated SBPH (Fig 9), indicating that LsTUB knockdown does not alter the feeding behavior of SBPH. As mentioned above, plants fed on by dsLsTUB-treated viruliferous SBPH showed a lower infection rate (Table 3). Thus, the decrease in RSV transmission efficiency in dsTUB-treated viruliferous SBPH can be attributed to reduced RSV accumulation and transmission in the insect.

## Discussion

The intracellular tubulin family is highly-conserved [38] and consists of α, β and γ subfamilies; the α- and β-tubulin subunits are highly heterogeneous and numerous isotypes exist with different expression patterns [39]. The α- and β-tubulin heterodimers interact laterally and longitudinally to form microtubules in the cytoskeleton [40, 41], and function in intracellular transport and cell division in eukaryotic organisms [42]. The tubulins and formed microtubules are also utilized by Chikungunya virus and Suid alphaherpesvirus 1 for viral internalization [43, 44]; whereas Human immunodeficiency virus-1, dengue 2 virus, Vaccinia virus and John Cunningham virus use them for trafficking [45, 46, 47, 48]. Furthermore, tubulins and microtubules are used by Human parainfluenza virus type 3 for viral replication [49], and Japanese encephalitis virus and West Nile virus use them for viral assembly [50, 51]. SBPH was shown to contain two or more genes encoding both α- and β-tubulin, and additional full-length sequence of the β-3 tubulin gene has also been identified [52]. However, no prior reports exist documenting a role for SBPH tubulin in virus transmission. In the present study, we cloned the gene encoding *LsTUB* (Fig 1) and showed that it was highly expressed in RSV-infected salivary glands of SBPH (Fig 2). Subsequent experiments revealed that suppression of *LsTUB* expression resulted in attenuated spread of RSV from midgut to salivary gland and reduced RSV transmission to rice plants (Fig 8, Table 2). These findings document a novel function for LsTUB in enabling RSV to overcome the midgut and salivary gland barriers of SBPH, leading to the dissemination of the virus to other organs in the insect vector.

In insect vectors, the interaction between host tubulin and viral proteins has been associated with viral transport. Recently, α-tubulin in the leafhopper *Nephotettix cincticeps* was shown to interact with nucleorhabdovirus matrix (M) protein from rice yellow stunt virus (RYSV), which impacted viral transport through neural tissue [53]. Our results showed that knockdown or overexpression of *LsTUB* did not alter RSV replication in insect cells (Fig 7). However, when LsTUB-NS3 binding was disrupted, the spread of RSV from the initially-infected midgut cells to salivary glands was inhibited (Fig 8). Thus, we suggest that LsTUB-NS3 binding facilitates RSV transport in SBPH cells, which is similar to the interaction of α-tubulin and the M protein from RYSV [53]. Meanwhile, the distribution analysis revealed the co-localization of LsTUB with NS3 or CP in SBPH cells (Figs 3 & 4). Although there is no direct interaction between NS3 and CP, the two proteins aggregated and formed inclusion bodies in Sf9 cells [54]. Thus, we speculate that LsTUB and NS3 associate with CP via an unknown mechanism. Taken together, our results suggest that LsTUB-generated microtubules bind to NS3 and recruit CP to facilitate virion transport through SBPH cells, thereby overcoming organ barriers; however, the actual mechanism *in vivo* warrants further exploration.

RSV requires NS3 silencing suppressor activity to counter host defenses, which facilitates systemic infection of both rice and SBPH [55, 56]. A recent report demonstrated that RSV NS3 can hijack the 26S proteasome by interacting directly with the SBPH RPN3 protein [55], suggesting. RSV NS3 may have additional functions that have not yet been elucidated. Our observations using Y2H and GST pull-down assays showed that NS3 interacts with LsTUB, and the binding site of NS3 to LsTUB was required for NS3 self-interaction and silencing suppressor activity (Fig 4). Furthermore, our results confirm that, in addition to silencing suppressor activity, the binding site of NS3 to LsTUB also plays a critical role in RSV transmission (Fig 6). Thus, RSV NS3 interacts with LsTUB to enhance dissemination of the virus in the insect vector.

Multiple reports have demonstrated that circulative-propagative viruses establish infections in insect midgut [32, 57], which is the initial barrier. These viruses then disseminate into other organs until they reach the final barrier, e.g. the salivary glands [24, 58]. Based on previous reports, two SBPH components are known to interact with the RSV CP to help the virus overcome the midgut barrier. One interaction consists of LsST6-CP binding, which mediates viral entry into midgut epithelial cells [26]. Another is GPS2-CP binding, an interaction that activates the SBPH JNK (c-Jun N-terminal kinase) signaling pathway in the midgut, which is beneficial to viral replication [32]. With respect to the salivary gland barrier, only CPR1- or GPS2-CP binding were reported to facilitate viral infection in the salivary glands [31, 32]. The spread of RSV in SBPH is obviously complex and requires multiple components. In this study, the interaction between LsTUB and NS3 facilitated RSV infection of midgut and salivary glands, which results in successful virus transmission to rice plants. Our findings thus complement and improve overall knowledge of the mechanistic basis of viral transmission in the SBPH vector; however, whether the LsTUB-NS3 interaction functions differently in the midgut vs. salivary glands requires further study.

Plant virus transmission is closely associated with the feeding behavior of insect vectors; therefore, monitoring the feeding process of dsRNA-treated SBPH can reveal the impact of dsRNA on feeding behavior and subsequent transmission to rice plants. EPG recordings have been used to investigate stylet penetration behavior in hemipteran insects [37, 59]. In the present study, the RSV infection rate in dsTUB-treated SBPH was significantly lower than the control group; however, the feeding behaviors of dsTUB-treated and control SBPH were not significantly different. Thus, the decrease in RSV transmission rate was not the result of altered feeding behavior, but was instead attributed to inefficient crossing of midgut and salivary gland barriers due to dsTUB treatment.

In summary, our results indicate that LsTUB helps RSV overcome the midgut and salivary gland barriers and enhances horizontal transmission of the virus. This conclusion is supported by immunofluorescent monitoring of LsTUB and RSV in midgut and salivary glands and by Y2H and pull-down assays with LsTUB and NS3 *in vitro*. Silencing of LsTUB expression by RNAi also reduced NS3 levels and consequently reduced viral dissemination into midgut and salivary glands, which ultimately reduced re-inoculation into the plant. These insights provide a better understanding of the interaction between plant viruses and vectors and may ultimately reveal new avenues for therapeutic intervention.

## Materials and methods

### Insects

Non-infected (RSV-free) and viruliferous strains of *L*. *striatellus* were originally collected from Jiangsu Province, China, and were maintained in the laboratory for eight years. Both non-infected and viruliferous SBPHs were reared independently on 2–3 cm seedlings of rice cv. Wuyujing 3 in glass beakers containing soil at a depth of 3–5 cm. Plants were maintained in a growth incubator at 25 ± 1°C, with 80 ± 5% RH and a 12-h light-dark photoperiod. Under these environmental conditions, SBPH development took 7 days for the egg stage, 15–18 days for the nymph stage, and 10–12 days for the adult stage. During the 30–37 day developmental period, both non-infected and viruliferous strains were transferred to fresh seedlings every 10–12 days for sufficient nutrition. To ensure that insects were viruliferous, individual female insects were allowed to feed independently. Parents and resulting offspring were collected in two-week intervals and analyzed via Dot-ELISA using monoclonal RSV CP-specific antibodies (anti-CP, 1:500) [60]. Highly viruliferous colonies were retained and used in subsequent studies.

To obtain viruliferous SBPHs, non-infected second instar nymphs were exposed for two days to RSV-infected rice plants with visible symptoms. Thereafter, RSV-positive individuals were identified via Dot-ELISA.

### Virus

More than 50 viruliferous SBPHs were placed into a glass beaker containing rice seedlings (2–3 cm high), and the RSV-infected rice plants were then cultivated until symptoms appeared. The RSV-infected leaves were collected and stored at −80˚C in the laboratory until RSV purification.

### Cloning of *LsTUB*

Total RNA was isolated from 15–20 SBPHs using TRIzol reagent according to the manufacturer’s protocol (Invitrogen). The quality and concentration of total RNA were determined by spectrophotometry (NanoDrop, Thermo Scientific). The extracted RNA (500 ng) was subsequently used for reverse transcription in a 10 μl reaction with the PrimeScript RT reagent kit and gDNA Eraser as recommended by the manufacturer (Takara, Dalian, China). Based on the α-tubulin mRNA sequence downloaded from NCBI (GenBank accession no. AY550922.1), 5' and 3' RACE were conducted to obtain the full-length transcript of α-tubulin using 5’- and 3’-RACE kits (Takara). The predicted LsTUB protein sequence was subjected to Blast analysis using DNAman software (LynnonBiosoft, USA), and domains of the predicted protein were deduced using SMART (http://smart.embl-heidelberg.de/) [61].

### Tissue collection

SBPH adults and nymphs were anesthetized with CO_2_ for 1–3 min, and then placed into a culture dish (35 mm diameter). First, forelegs were removed from the coxa-trochanter joint using forceps (WPI, Sarasota, FL, USA), and hemolymph droplets were collected to avoid contamination by the fat body [62]. The remaining SBPHs were then dissected in prechilled and sterile PBS buffer (pH 7.4, 10 mM) to obtain midguts and salivary glands. All tissues were washed three times in sterile PBS buffer to remove other tissues, proteins, and surface contaminants.

### Real-time qRT-PCR

To measure *LsTUB* expression levels and RSV copy number equivalents in SBPH, total RNA was isolated from 50 whole bodies, 50 midgut or 100 salivary glands of SBPH adults (female / male ratio = 1:1) using the TRIzol Total RNA Isolation Kit (Takara, Dalian, China). Total RNA concentrations were quantified, and first-strand cDNA was synthesized as described above. The primers (S2 Table) used for detecting RSV titers were designed based on RSV CP-specific nucleotide sequences. Similarly, *LsTUB* and *LsActin* (control) primers (S2 Table) were designed based on *LsTUB* and *LsActin* sequences, respectively. qRT-PCR was conducted using a CFX96 Real-Time PCR Detection System (Bio-Rad, Hercules, CA, USA) and SYBR Premix Ex Taq (Takara, Dalian, China) as follows: denaturation for 3 min at 95°C, followed by 40 cycles at 95°C for 10 s, and 60°C for 30 s. Relative expression levels for triplicate samples were calculated using the ΔΔCt method, and expression levels of target genes were normalized to the SBPH *Actin* gene. Three technical repeats were performed for each of the three biological replicates.

### Antibodies

The mouse polyclonal antibody against the RSV CP peptide was produced by HuaAn Biotechnology Co., Ltd (HuaBio, Hangzhou, China). The rabbit anti-RSV NS3 was kindly provided by Dr. Kun Zhang (Yangzhou University). Due to highly conserved α-2-tubulin, the rabbit monoclonal anti-α-TUB antibody (EP1332Y, Abcam, UK) was used to detect LsTUB in SBPHs. The following antibodies were obtained from the sources indicated: goat anti-mouse IgG HRP conjugate (cat. CW0102S, Cwbiotech, China), goat anti-rabbit IgG HRP conjugate (cat. CW0103S, Cwbiotech, China), Alexa Fluor 488-labeled goat anti-mouse IgG (cat. 115-545-003, Jackson ImmunoResearch Laboratories, USA), Alexa Fluor 555-labeled donkey anti-rabbit IgG (cat. ab150074, Abcam, UK), rabbit polyclonal anti-His tag (cat. 2365, Cell Signaling Technology, USA), and rabbit polyclonal anti-GAPDH antibody (cat. ab157156, Abcam, UK). DAPI (4’,6-diamidino-2-phenylindole) was from Sigma (cat. 28718-90-3, Sigma, USA).

### Western blotting

Whole body (50), midgut (50) and salivary gland (100) samples of adults (female / male ratio = 1:1) were collected and lysed with TRIzol reagent (ThermoFisher, USA) or Cell Lysis reagent (Beyotime, China) for protein extraction according to the manufacturer’s protocols. After adding 6× SDS loading buffer, 50 μg protein samples were boiled for 10 min. The proteins were separated by 8–12% SDS-PAGE and transferred onto PVDF membranes. Blots were probed with the following antibodies: anti-LsTUB (1:1000 dilution), anti-RSV CP (1:1000 dilution), anti-RSV NS3 (1:500 dilution), or anti-GAPDH (1:2000 dilution). Immuno-reactive bands were detected using a goat anti-rabbit/goat anti-mouse IgG-conjugated HRP antibody and a goat anti-mouse IgG-conjugated HRP antibody (Proteintech, USA) at a 1:5000 dilution. Western blots were imaged with a Chemiluminescence Detection Kit (Bio-Rad, Hercules, CA, USA) and the Molecular Imager ChemiDo XRS System (Bio-Rad). Triplicate samples were performed for each experiment and the relative intensities of protein expressions were calculated using Image lab 5.2.1 software (Bio-Rad).

### RNA interference (RNAi)

The coding sequences of *LsTUB* and GFP were cloned into pMD19-T vectors (Takara, Japan). The primers for dsGFP and dsTUB amplification are listed in S2 Table. Using the cDNA templates obtained above, dsRNAs were synthesized using the T7 RiboMAX Express RNAi System kit as recommended by the manufacturer (Promega, USA). A membrane feeding approach was used to introduce dsRNAs into SBPHs as described previously [57, 63, 64]. Briefly, second instar nymphs of viruliferous SBPH were maintained on a mixed diet containing 0.5 mg/ml dsRNAs for four days via membrane feeding and then transferred to healthy rice seedlings. To investigate the RNAi effect of dsLsTUB on viral spread in SBPH, a microinjection method was used as previously reported [26, 28]. Third instar non-infected nymphs were microinjected with 23 nl dsLsTUB (2.5 μg/μl) or dsGFP (2.5 μg/μl) using an UMP3-2 UltraMicroPump (UMP3) with SYS-Micro4 Controller (WPI, FL, USA), followed by a two-day acquisition period on RSV-infected rice plants. All individuals were transferred and maintained on healthy rice seedlings until evaluation immunofluorescence, qRT-PCR or Western bolt analysis. The effects of dsRNA on *LsTUB* expression was investigated by qRT-PCR.

### Immunofluorescence microscopy

More than 30 SBPH adults were maintained on rice plants for seven days after RNAi treatment and dissected to obtain midgut and salivary glands. The dissected samples were fixed with 4% paraformaldehyde for 1 h. Samples were then blocked using 10% fetal bovine serum at ambient temperature for 2 h. Samples were incubated for 16 h at 4°C with preimmune serum and anti-LsTUB or anti-RSV CP antibody at 1:500 dilution before incubation with the following secondary antibodies: Alexa Fluor 555- or Alexa Fluor 488-labeled secondary goat anti-rabbit IgG. Samples were then washed three times in PBS, and stained with 100 nM DAPI and CM-Dil (Sigma-Aldrich, USA) for 2 min at room temperature. Fluorescence was observed with a Leica TCS SP8 STED confocal microscope (Leica, Germany).

### Yeast two-hybrid assay

Yeast two-hybrid assays were conducted using protocols supplied with the Yeastmaker Yeast Transformation System 2 (Takara-Clontech, USA). Briefly, the cDNA library of RSV was cloned as prey in plasmid vector pGADT7 using the Easy Clone cDNA library construction kit (Dualsystems Biotech), and a full-length *LsTUB* was cloned as bait in pGBKT7. Positive clones were selected on SD quadruple-dropout (QDO) medium (SD/-Ade/-His/-Leu/-Trp), and interacting prey constructs were recovered and sequenced. To distinguish positive from false-positive interactions, we co-transformed empty pGADT7 and pGBKT7 into yeast strain Y2HGold. ß-galactosidase activity was detected with the HTX Kit (Dualsystems Biotech).

### GST pull-down assay

*LsTUB* cDNA fragments were amplified and cloned into pGEX-3X as glutathione-S-transferase (GST) translational fusions. Recombinant proteins were produced in *Escherichia coli* strain BL21 and purified. For pull-down assays, viruliferous SBPH extract (1 mg), immobilized glutathione-Sepharose beads (200 μl) and GST-LsTUB protein (500 μg) were added to 1 ml of pull-down buffer (50 mM Tris, 150 mM NaCl, 0.1% Triton X-100, 1 mM PMSF, 1% protease inhibitor cocktail [pH 8.0]), and then incubated at 4°C for 16 h. Similarly, insect extracts were incubated with GST protein as a negative control. Beads were washed four times with pull-down buffer, and retained proteins were released by adding 2× loading buffer and incubating for 5 min at 95°C. Proteins were then separated by SDS–PAGE and detected using anti-GST (Cusabio, China) and anti-NS3 antibodies.

### RNA silencing suppression activity

*LsTUB*, RSV *NS3* and two mutant forms of *NS3* were amplified from pGBKT7-LsTUB, pGADT7-RSV NS3, pGADT7- RSV NS3 mut ^SFL^74-76^AAA^ and RSV NS3 mut ^IWI^80-82^AAA^ respectively. The products were inserted into pGEM-T (Promega, USA) and sequenced. Plasmids were then digested with *Bam*HI/*Sal*I and inserted into the binary vector pBin438, a plasmid expressing 35SGFP35S-GFP [65]. Plasmids expressing the *Cymbidium ringspot virus* P19 gene (P19), GFP, and an inverted repeated sequence of GFP (dsGFP) were also used in this experiment. Constructs were individually electroporated into *A*. *tumefaciens* C58C1. For co-inoculation assays, *A*. *tumefaciens* strains were grown to OD_600_ = 0.6–0.8, and one-month-old *N*. *benthamiana* leaves were spot-inoculated with *A*. *tumefaciens* harboring LsTUB, GFP, dsGFP, NS3 or the NS3 mutants using 1 ml syringes. GFP silencing in leaf tissue was assessed under UV light at 3–4 days post inoculation, and leaves were photographed with a Nikon digital camera (model D800).

### LsST6-transfected Sf9 cell line

The *S*. *frugiperda* Sf9 insect cell line was provided by Prof. Fei Ma (Nanjing Normal University) and incubated at 27˚C in Sf-900 III SFM medium (Gibco, USA) containing 5% newborn calf serum. *LsST6* was amplified based on the SBPH sequence deposited in NCBI (GenBank accession no. MG589412). The PCR product containing *LsSt6* was cloned as a *Bam*HI/*Xba*I fragment in pFastBacHTB (Invitrogen, USA) and introduced into Sf9 cells to generate the LsST6-Sf9 cell line as described previously [26].

### Transfection and RSV infection of Sf9 cells expressing *LsST6*

A baculovirus expression system (Bac-to-Bac, Invitrogen, USA) was used to overexpress *GFP* and *LsTUB* in LsST6-Sf9 cells. The LsST6-Sf9 cells were transfected with 0.5 mg/ml dsGFP or dsLsTUB and Cellfectin II reagent (Invitrogen, USA) for 24 h as described previously [26]. At 48 h post-transfection, RSV particles (1.5 μg/μl) were added to the Sf9 cells as described [26], and RSV-infected Sf9 cells were collected at 1, 6, 12 and 24 h post-infection and washed five times in double-distilled water. The Sf9 cell lines were fixed and prepared for confocal microscopy. Total RNA was also extracted from these cells using TRIzol Total RNA Isolation Kit (Takara, Dalian, China) for qRT-PCR, and total protein was extracted for Western blot analysis. Each well of the plate was considered to be a single replicate, and each treatment contained three replicates.

### RSV acquired from infected plants spread in SBPHs

The SBPHs can acquire and transmit RSV at almost all stages of development [15]. The latent period of RSV in SBPHs ranges from 3 to 10 days [26, 66]. No significant sexual dimorphism in regard to virus acquisition efficiency was observed. However, in general, females were more efficient vectors than males in terms of RSV transmission [15, 67]. More than 400 3^rd^ instar nymphs of SBPH were microinjected with dsGFP or dsLsTUB, followed by a two-day acquisition period on RSV-infected rice as described above. At 1, 3, 5, and 7 day post-initial access to diseased plants (padp), 100 SBPHs were collected and dissected to obtain midguts, hemocytes, and salivary glands for RSV detection using RSV NS3 antibody and the number of infected organs was recorded.

### Electrical penetration graph (EPG) recording and data analysis

More than 50 nymphs from RSV-infected SBPH strains were reared for seven days on artificial liquid diets [68] supplemented with one of the following: dsGFP, dsLsTUB, or no dsRNA (control). After a 30-min starvation, the mesonotum of *L*. *striatellus* was affixed with a gold wire (20 μm diameter, 2–3 cm long) using a soluble conductive adhesive. Each *L*. *striatellus* individual was then connected to an eight-channel EPG recorder (Model: CR-8 DC-EPG I). Nymphs were placed on the culms of rice seedlings (three-leaf stage) in Ferrari insect cages; activity was recorded for 8 h in a greenhouse maintained at 25–26°C, with 60±5% RH. Nymphs were removed at the end of the 8 h period and analyzed for RSV by Dot-ELISA. If an insect tested virus-free, the data were considered invalid. Each treatment contained 20–30 replicates, and all recorded signals were analyzed.

### RSV transmission efficiency

#### The transmission efficiency of SBPH acquiring RSV from infected plants

More than 100 SBPH individuals acquired RSV from infected plants were microinjected with dsGFP or dsLsTUB as described above. SBPH individuals were kept independently on non-infected rice seedlings for 2 days. Each individual was then removed and analyzed by dot-ELISA to confirm viral infection. Non-infected (non-viruliferous) individuals and corresponding seedlings were eliminated from the experiment. The remaining rice seedlings colonized by RSV-viruliferous insects (25–30 plants/treatment) were incubated another 10–15 days. RSV infection of rice seedlings was confirmed by qRT-PCR using RSV CP primers (S2 Table).

#### The transmission efficiency of SBPH acquiring RSV from parents

Following the EPG analysis above, rice seedlings fed on by RSV-infected SBPH were also incubated an additional 10–15 days to ensure virus propagation. RSV infection of rice seedlings used in EPG analysis was determined by qRT-PCR as described above.

#### Evaluation of RSV transmission rates

The number of infected rice seedlings was recorded and calculated to determine transmission rates; rice seedlings colonized by dsGFP-treated or untreated SBPH were considered controls. Each seedling was considered to be one replicate, and each treatment had 25–30 replicates. Transmission rates were calculated as follows: transmission rate (%) = (number of infected seedlings/total number of seedlings) × 100. A *χ^2^* test was performed with SPSS statistical software [69] to detect differences between treatments.

## Supporting information

S1 Fig Western blot analysis of LsTUB and RSV NS3 protein in non-infected and viruliferous SBPH. (TIF)

S2 Fig Yeast cotransformants were incubated on DDO (SD/–Leu/–Trp) and QDO (SD/–Ade/–His/–Leu/–Trp plus X-α-Gal) at 28°C for 4 days. (TIF)

S3 Fig The interaction between LsTUB and other viral proteins detected by yeast two-hybrid assay. (TIF)

S4 Fig Relative levels of *LsTUB* mRNA after RNAi-mediated knockdown. *LsTUB* expression in untreated, and dsGFP- or dsTUB-treated SBPH. *LsTUB* expression was evaluated by qRT-PCR and normalized relative to GAPDH transcript levels. Values represent means ± SE. Significance was evaluated by *t*-test analysis, and *** is significant at *P*<0.001. Treatments were replicated three times.
(TIF)

S5 Fig Relative intensity of LsTUB and RSV NS3 protein levels in dsTUB-treated midgut and salivary glands. Significance was evaluated by *t*-test analysis: *, significant at *P*<0.05; **, significant at *P*<0.01; and ***, significant at *P*<0.001. Treatments were replicated three times.
(TIF)

S6 Fig The mRNA expression levels of LsTUB and LsST6 in LsST6-tranfected Sf9 cells containing dsLsTUB or LsTUB. (A) The mRNA levels of LsTUB (B) and LsST6 in dsLsTUB-treated and LsTUB-overexpressing Sf9 cells.
(TIF)

S7 Fig qRT-PCR analysis of RSV loads in midguts from dsGFP- or dsTUB-treated SBPH at 3 days padp. (TIF)

S1 Table Percentage of RSV infected tissues of SBPH as revealed by immunofluorescence microscopy (only typical, representative images were taken into consideration). (DOCX)

S2 Table Primers used in this study. (DOCX)
