# Supplementary figures and images for: The α-tubulin of Laodelphax striatellus mediates the passage of rice stripe virus (RSV) and enhances horizontal transmission
Source: PLoS Pathog. 2020 Aug 20;16(8):e1008710. doi: 10.1371/journal.ppat.1008710 (PMC7446811; doi:10.1371/journal.ppat.1008710)

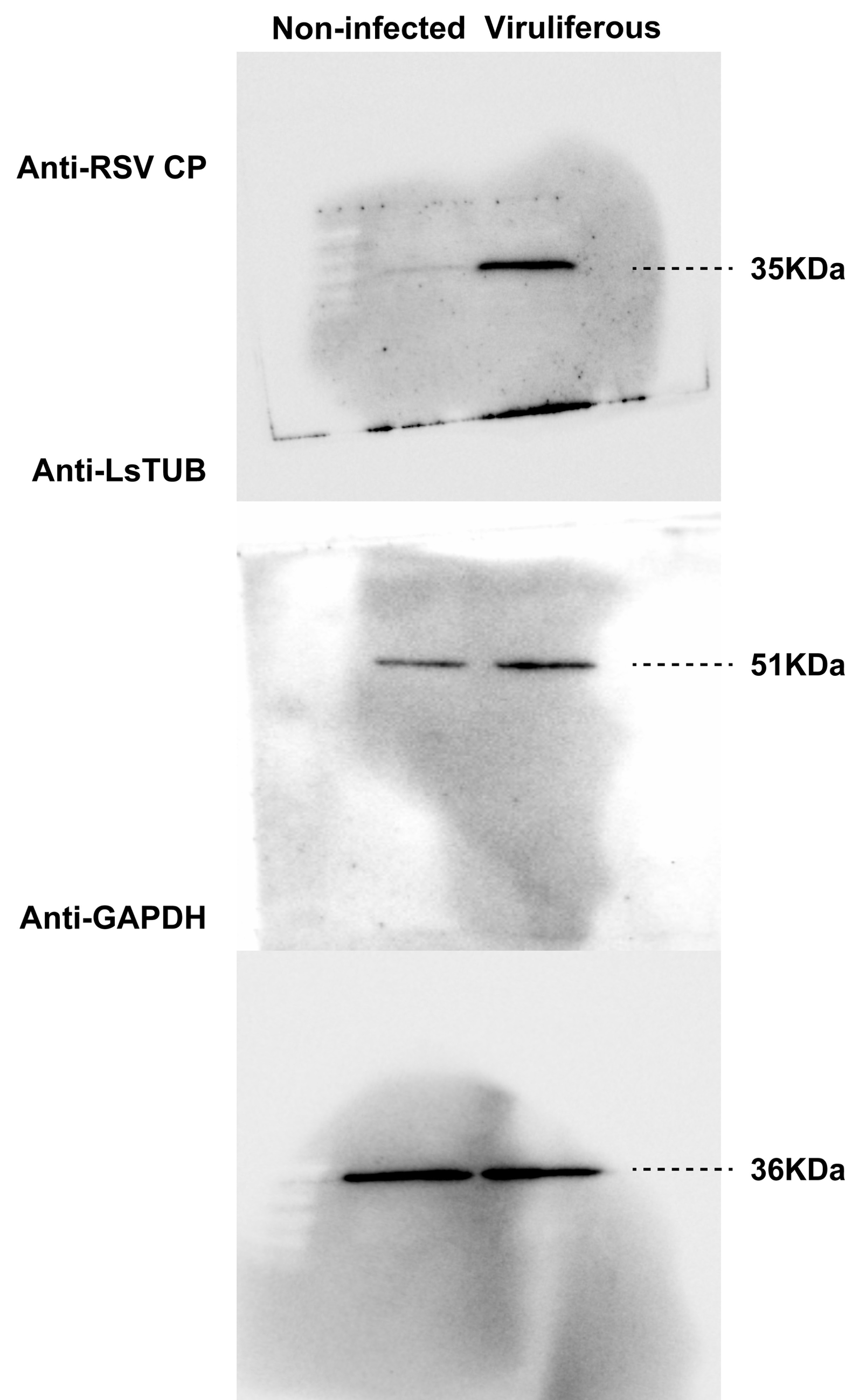

Supplement: S1 Fig — (TIF) [file ppat.1008710.s001.tif]

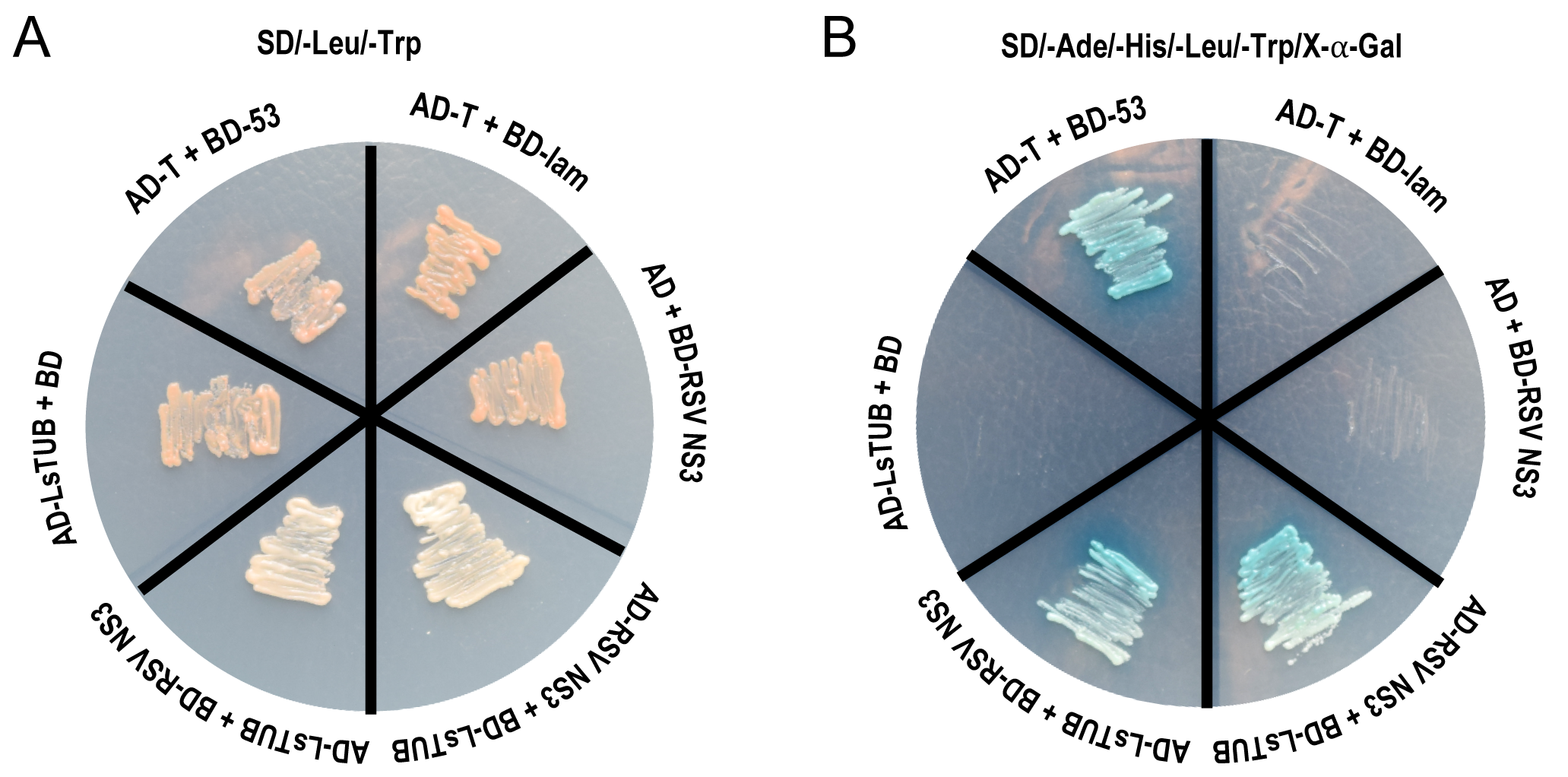

Supplement: S2 Fig — (TIF) [file ppat.1008710.s002.tif]

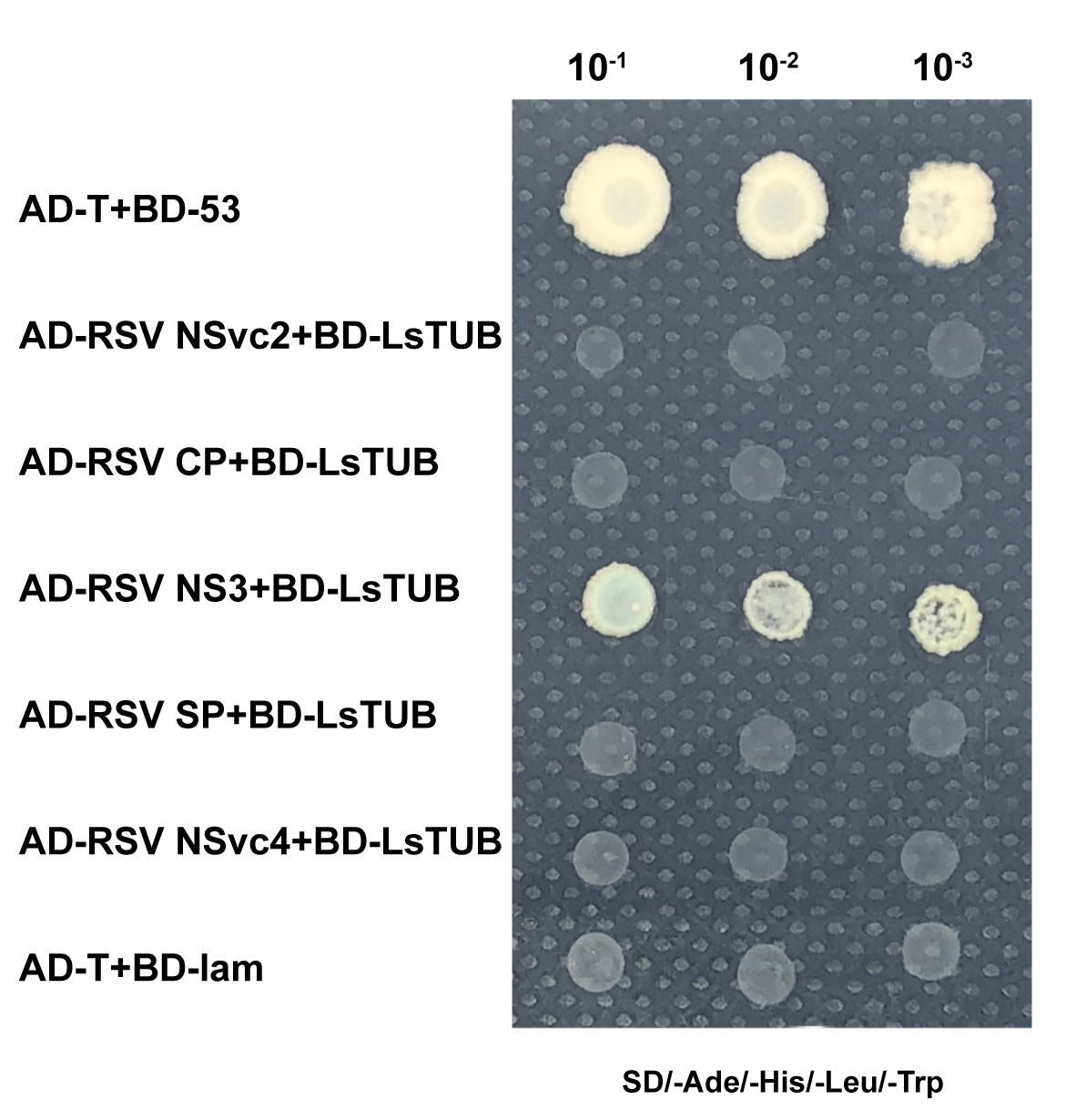

Supplement: S3 Fig — (TIF) [file ppat.1008710.s003.tif]

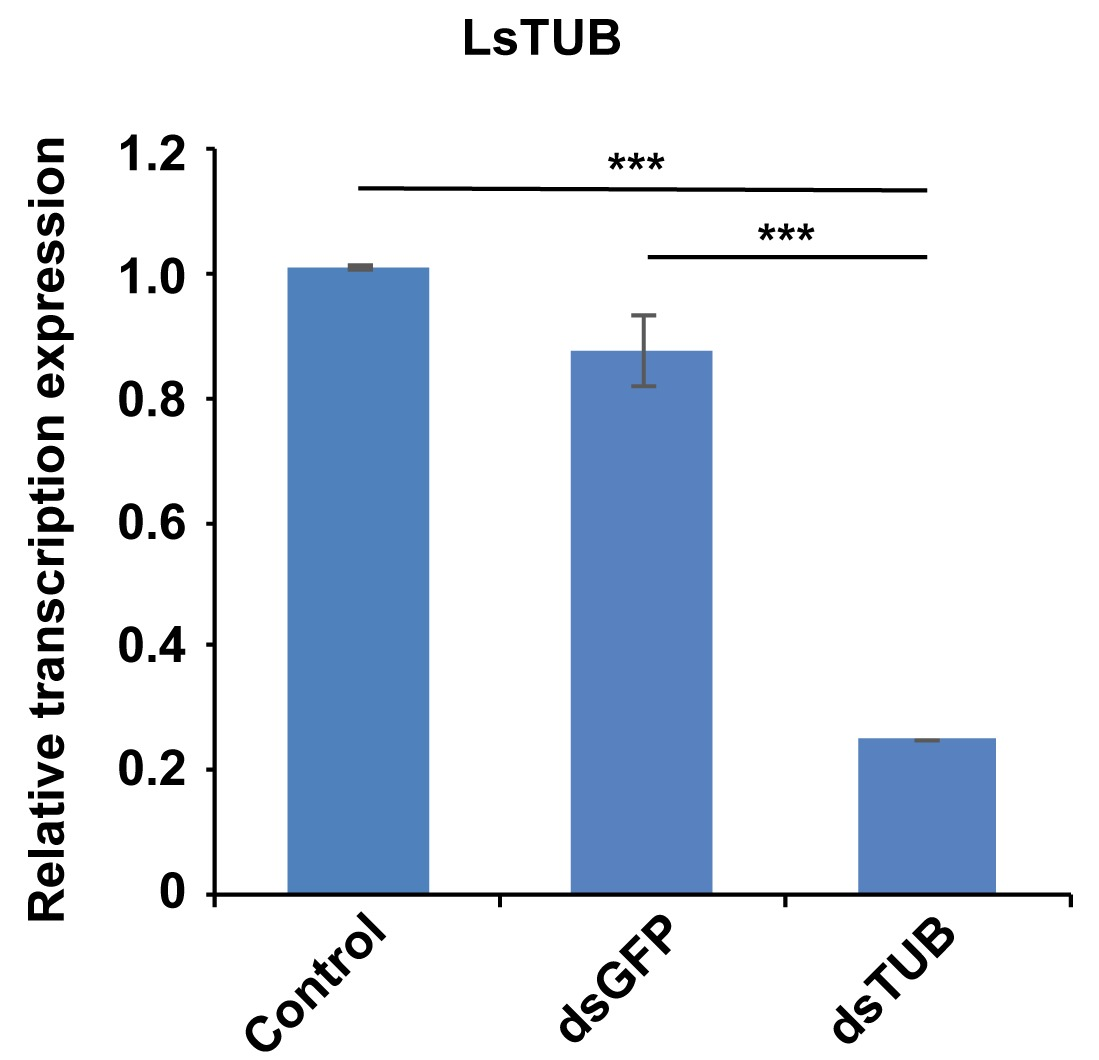

Supplement: S4 Fig — LsTUB expression in untreated, and dsGFP- or dsTUB-treated SBPH. LsTUB expression was evaluated by qRT-PCR and normalized relative to GAPDH transcript levels. Values represent means ± SE. Significance was evaluated by t-test analysis, and *** is significant at P<0.001. Treatments were replicated three times. (TIF) [file ppat.1008710.s004.tif]

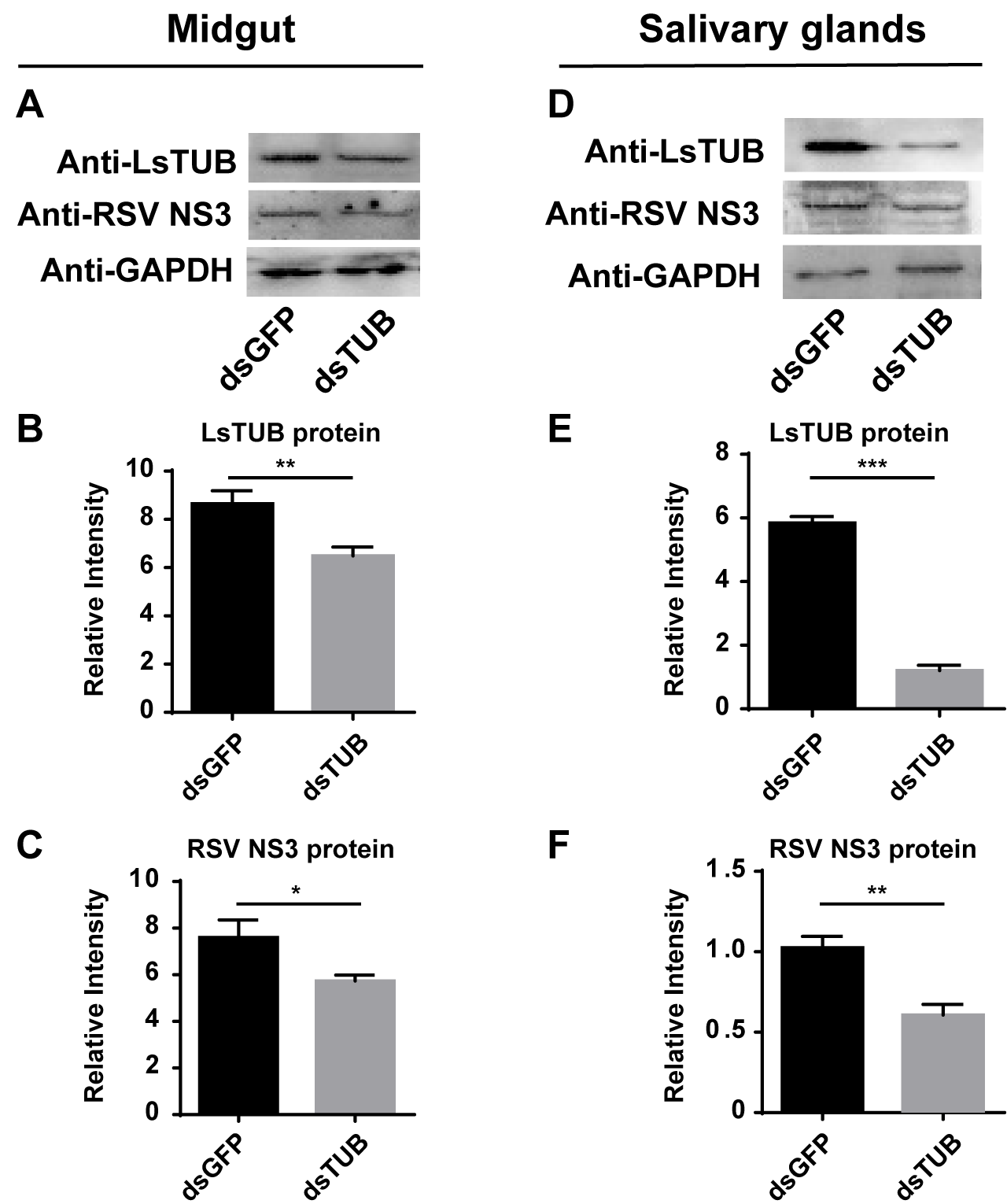

Supplement: S5 Fig — Significance was evaluated by t-test analysis: *, significant at P<0.05; **, significant at P<0.01; and ***, significant at P<0.001. Treatments were replicated three times. (TIF) [file ppat.1008710.s005.tif]

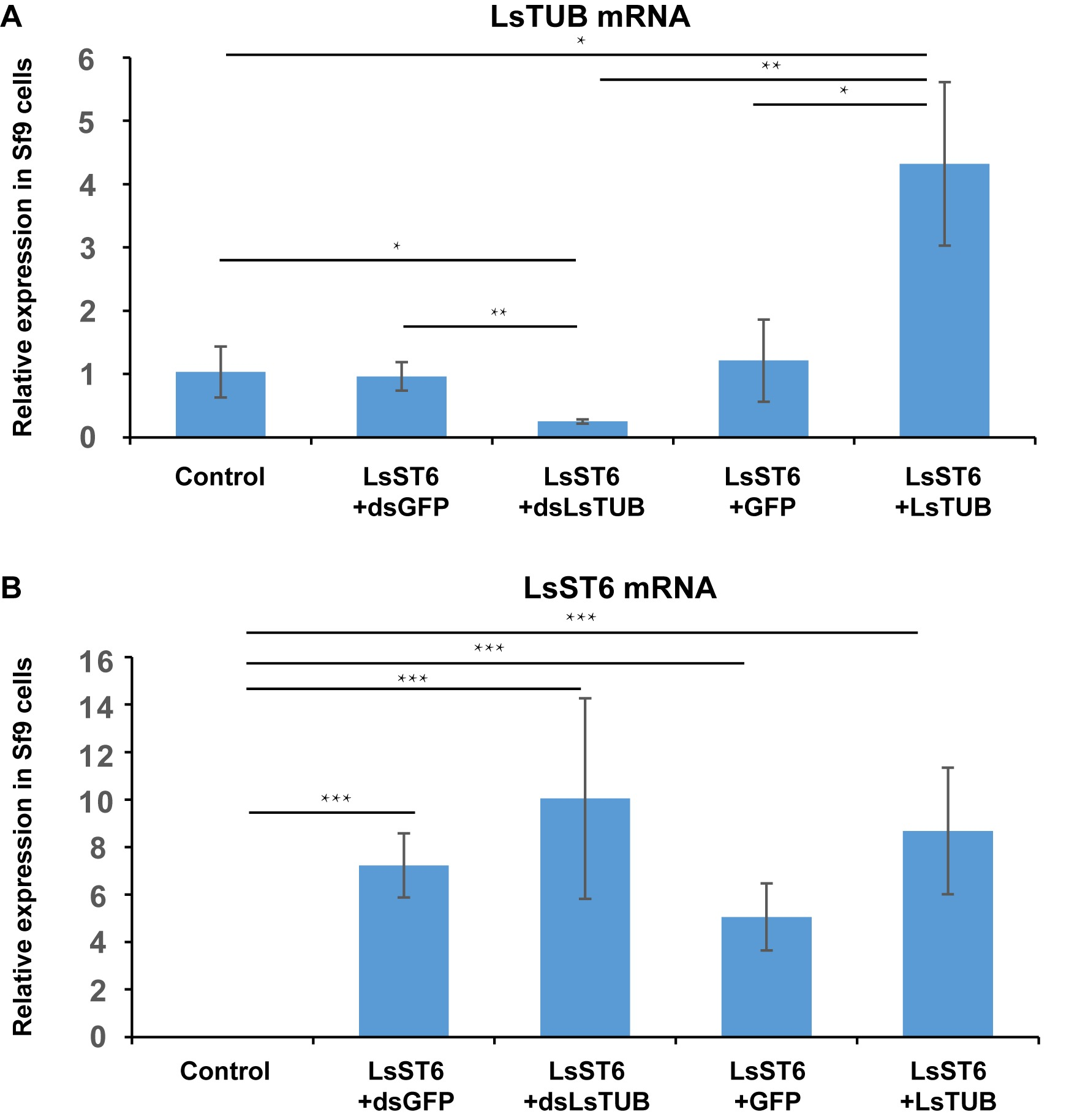

Supplement: S6 Fig — (A) The mRNA levels of LsTUB (B) and LsST6 in dsLsTUB-treated and LsTUB-overexpressing Sf9 cells. (TIF) [file ppat.1008710.s006.tif]

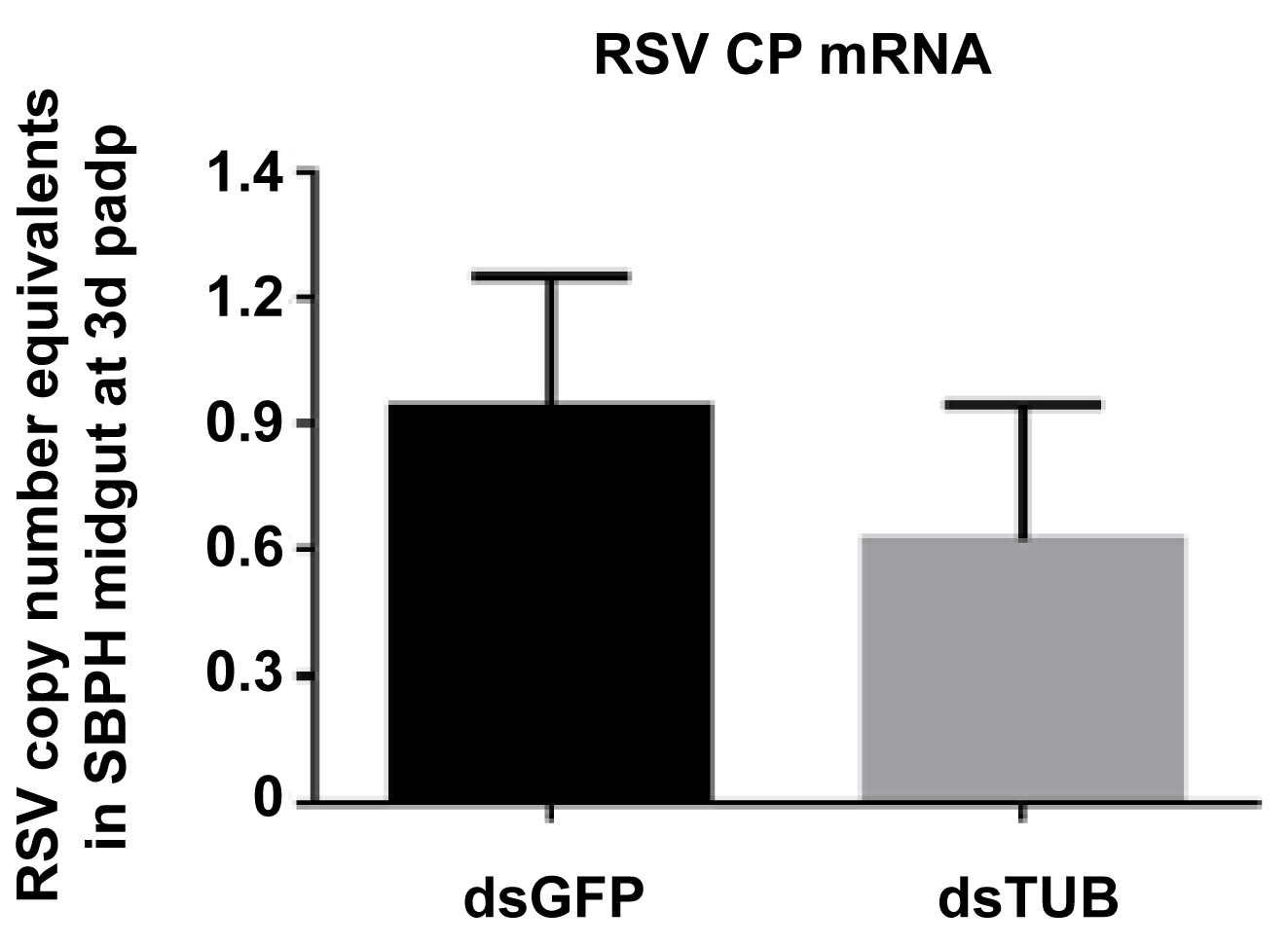

Supplement: S7 Fig — (TIF) [file ppat.1008710.s007.tif]
