# Supplementary material for: The α-tubulin of Laodelphax striatellus mediates the passage of rice stripe virus (RSV) and enhances horizontal transmission
Source: PLoS Pathog. 2020 Aug 20;16(8):e1008710. doi: 10.1371/journal.ppat.1008710 (PMC7446811; doi:10.1371/journal.ppat.1008710)
Supplement: S1 Table — (DOCX) [file ppat.1008710.s008.docx]

**S1 Table. Percentage of RSV infected tissues of SBPH as revealed by immunofluorescence microscopy (only typical, representative images were taken into consideration).**

| Tissues examined | Treatment | Number of viruliferous SBPH tissues positive | Number of viruliferous SBPH tissues negative | Percentage of viruliferous SBPH tissues positive (%) |
| --- | --- | --- | --- | --- |
| Midgut | dsGFP | 30 | 0 | 100.0 |
|  | dsTUB | 22 | 8 | 73.3 |
| Salivary gland | dsGFP | 28 | 2 | 93.3 |
|  | dsTUB | 19 | 11 | 63.3 |
