# Supplementary material for: The α-tubulin of Laodelphax striatellus mediates the passage of rice stripe virus (RSV) and enhances horizontal transmission
Source: PLoS Pathog. 2020 Aug 20;16(8):e1008710. doi: 10.1371/journal.ppat.1008710 (PMC7446811; doi:10.1371/journal.ppat.1008710)
Supplement: S2 Table — (DOCX) [file ppat.1008710.s009.docx]

**S2 Table. Primers used in this study.**

| Gene name | primers | Sequences |
| --- | --- | --- |
| *RSV CP* | q-RSV-F | TGAAAGTGGCGGCTGGAA |
|  | q-RSV-R | CCACCGAGGACACTATCCCATA |
| *LsActin* | Actin-F | GTCTCACACACAGTCCCCATCTATG |
|  | Actin-R | TCGGTCAAGTCACGACCAGC |
| *GFP* | GFP-F | AAGGGCGAGGAGCTGTTCACCG |
|  | GFP-R | CAGCAGGACCATGTGATCGCGC |
|  | T7GFP-F | TAATACGACTCACTATAGGGAAGGGCGAGGAGCTGTTCACCG |
|  | T7GFP-R | TAATACGACTCACTATAGGG CAGCAGGACCATGTGATCGCGC |
| *LsTUB* | ds-TUB-1-F | CCAACAACTACGCCAGAGG |
|  | ds-TUB-1-R | CGAAGTGAAGCCAGAGCC |
|  | T7ds-TUB-2-F | TAATACGACTCACTATAGGG TGCTCCTCAGGTATCAACAG |
|  | T7ds-TUB-2-R | TAATACGACTCACTATAGGG TCGTGGTAGGCTTTCTCG |
|  | q-TUB-F  q-TUB-R | CCAACAACTACGCCAGAGG  CGAAGTGAAGCCAGAGCC |

**Note:** RSV CP, encodes capsid protein of RSV; *LsActin*, Actin of SBPH; and *LsTUB*, tubulin α-2 of SBPH.
